# THEORIES, MODELS, AND FRAMEWORKS IN IMPLEMENTATION SCIENCE IN THE CONTEXT OF REHABILITATION RESEARCH: A SCOPING REVIEW

**DOI:** 10.2340/jrm.v58.46016

**Published:** 2026-07-17

**Authors:** Maarit KARHULA, Hennariikka HEINIJOKI, Riitta SEPPÄNEN-JÄRVELÄ

**Affiliations:** Social Insurance Institution of Finland, Kela, Finland

**Keywords:** implementation science, knowledge translation, rehabilitation research, scoping review, theories, models and frameworks

## Abstract

**Objective:**

This review explores the use of implementation science theories, models, and frameworks in rehabilitation research by describing study characteristics and assessing how the applied theories, models, and frameworks align with study purposes.

**Methods:**

In the scoping review, peer-reviewed articles published in English between January 2010 and October 2023 were identified from 5 datbases. Eligible studies applied or were informed by implementation science theories, models, and frameworks in any rehabilitation cotext. Data extraction covered publication characteristics, target populations, setings, study designs, used theories, models, and frameworks, and study purposes. Descriptive statistics and deductive analysis were used to synthesize findings.

**Results:**

Of 2,277 citations, 121 articles met the inclusion criteria. Most of the articles focused on neurological and paediatric rehabilitation, with qualitative and mixed-methods designs predominating. A total of 34 theories, models, and frameworks were identified, mainly determinant frameworks and process models. Theories, models, and frameworks were primarily used to identify barriers and facilitators, evaluate implementation outcomes, and describe implementation processes. However, theories, models, and frameworks selection did not always align with the study purpose.

**Conclusion:**

The use of theories, models, and frameworks in rehabilitation research is increasing but remains limited. Their systematic and purposeful application is needed to enhance the usability and transferability of research findings, thereby bridging the research–practice gap, sustaining evidence-based interventions, and informing policy.

## LAY ABSTRACT

Implementation science approaches help translate research knowledge into everyday practice. This review examines how implementation science approaches have been used in rehabilitation research. Because rehabilitation interventions are often complex, different approaches are needed to address various aspects of implementation. Understanding how these approaches have been applied helps clarify the current state of implementation research in rehabilitation and identify future priorities. We reviewed articles identified through searches of multiple databases and extracted information from 121 studies. The approaches were most often used to identify factors that supported or hindered change, to examine how new practices were carried out, and to assess implementation-related outcomes. However, the selected approach did not always clearly align with the study’s actual aim. Although the use of implementation science approaches in rehabilitation research is growing, it remains limited. Clearer and more consistent use is needed to better connect research with clinical practice and support evidence-based rehabilitation services.

Rehabilitation is increasingly recognized as a complex intervention embedded within interconnected health, social, educational, and employment systems (1). Given the complexity and variability of rehabilitation, achieving a comprehensive definition that applies across contexts is challenging. The World Health Organization defines rehabilitation as “a set of interventions designed to optimize functioning and reduce disability in individuals with health conditions in interaction with their environment” (2). Wade (3) synthesized key characteristics from 2 review articles on rehabilitation concepts: rehabilitation is a process that integrates multiple actions into a coherent bundle; it is also a strategy aimed at optimizing functioning, social integration, autonomy, and quality of life. Definitions consistently emphasize that rehabilitation is person-centred. The general theory of rehabilitation presented by Wade (4) highlights that rehabilitation facilitates adaptation to illness while positioning the individual as an active agent. Goal-setting is central to this process, as goals are identified and prioritized by the person through shared decision-making with the rehabilitation team.

This systemic and multidimensional nature underscores the challenges of decision-making in rehabilitation, where multiple interacting factors must be considered to achieve individualized and effective outcomes. Consequently, contemporary rehabilitation practice increasingly emphasizes evidence-based approaches. Evidence-based rehabilitation integrates the best available research evidence with clinical expertise and client preferences to support nuanced, context-sensitive decisions. This approach demands not only awareness of relevant evidence but also effective communication, sound clinical judgement tailored to individual circumstances, and creative application when straightforward solutions are not feasible (5). Although the number of effectiveness studies has grown, they alone are insufficient to guide practice; their findings must be actively and systematically implemented in real-world settings (6). Introducing and sustaining new practices further requires drawing on implementation science theories, models, and frameworks (TMFs) (7).

Implementation research often addresses context-specific, practical questions within organizations, which can make findings difficult to generalize (8). To overcome this challenge, TMFs provide structured, tested concepts and mechanisms that explain implementation processes and outcomes (9). They enable the systematic evaluation of factors that influence implementation, support learning, and facilitate scaling interventions across diverse settings (10). By doing so, TMFs ensure that knowledge generated in one context can be transferred to similar environments, strengthening the rigour and transparency of implementation science.

Beyond theory, TMFs serve as practical tools for translating evidence into real-world rehabilitation practice. They offer structure for complex situations shaped by interacting factors at individual, organisational, and system levels, helping to identify barriers and facilitators and guiding the selection of effective strategies (11). Sustaining evidence-based practices remains a critical yet underexplored challenge (12), and TMFs can clarify the underlying mechanisms and inform strategies for long-term integration. Furthermore, by providing shared concepts and terminology, TMFs enable synthesis and comparison across studies, which is essential for advancing rehabilitation research and ensuring consistent uptake of evidence-based interventions (13, 14).

In implementation science, TMFs have been developed for distinct objectives. Because TMFs vary in focus and application, a wide range of them is required to address the diverse needs in the field of rehabilitation. Nilsen (15) proposed a classification of theoretical approaches that is widely used in implementation science. These approaches are grouped into 5 categories (process models, determinant frameworks, classic theories, implementation theories, and evaluation frameworks), each comprising several TMFs, and the categories serve 3 aims. The first aim is to describe and/or guide the process of translating research into practice, which is addressed using process models. The second aim is to understand and/or explain the determinants of implementation outcomes, which involves determinant frameworks, classic theories, and implementation theories. Finally, the third aim is to evaluate implementation, which is supported by evaluation frameworks. This classification provides a structured basis for selecting the appropriate TMFs, including those applied in rehabilitation research. In recent years, the use of TMFs within implementation science in rehabilitation research has expanded (16). Despite the availability of numerous frameworks, selecting the most suitable TMF for a given context remains challenging (17). Given the inherent complexity of rehabilitation interventions and settings, diverse frameworks are needed to address different aspects of implementation. Understanding how TMFs have been applied in rehabilitation research can clarify the current state of implementation science in this field and inform future priorities, for example whether to strengthen existing approaches or to emphasize areas that require further development.

## METHODS

This review aims to explore the use of implementation science TMFs in rehabilitation implementation research by describing publication trends and study characteristics and by assessing how applied TMFs align with study purposes. We used the scoping review methodology (18–20) to search, select, and synthesize knowledge. The process followed the 5 steps outlined by Arksey and O’Malley (18): identifying research questions, identifying relevant studies, selecting studies, extracting data, and data analysis and synthesis. Reporting was guided by the Preferred Reporting Items for Systematic Reviews and Meta-Analyses extension for Scoping Reviews (PRISMA-ScR) (21) (see Appendix S1). A protocol for this review was registered with OSF Registries (https://osf.io/8xhup).

### Identifying research questions

This study has 2 main objectives:

1. To describe the characteristics of rehabilitation studies using implementation science TMFs, including publication details, target populations, settings, and study designs.
2. To identify which TMFs have been applied and assess how their use aligns with the studies’ purposes.

### Identifying relevant studies

*Inclusion and exclusion criteria.* Studies were considered eligible if implementation science TMFs were applied. The classification proposed by Atzmon et al. (22) was used, categorizing TMF utilization as applied, informed by, or cited only. For inclusion, studies were required to either apply TMFs, indicating substantial integration of theoretical constructs into trial components, or be informed by TMFs, meaning limited influence, such as guiding qualitative analysis without measuring constructs. The cited-only category was excluded because mere citation does not demonstrate meaningful use of a theoretical framework in the implementation component.

Articles addressing the use of TMFs across all rehabilitation contexts were included, encompassing diverse settings and circumstances, such as direct rehabilitation services for clients in clinics, hospitals, rehabilitation centres, and communities, as well as service planning, organization, and recommendations or guidelines. No restrictions were imposed regarding the type of rehabilitation intervention or specific patient populations. This broad inclusion aimed to capture comprehensive insights into TMF selection, thereby enhancing the generalizability and relevance to various rehabilitation implementation scenarios. Studies conducted in any geographical location were eligible.

Furthermore, only studies published in peer-reviewed journals were included. Studies had to be published in English, and full-text articles needed to be available for review.

### Information sources

Searches were conducted in the following databases from January /2010 to October 2023: PubMed, CINAHL, SCOPUS, ScienceDirect, and SagePremier.

### Search strategy

The search strategies were as follows: (*i*) “translational research” OR “knowledge translation” OR “translational science” OR “translational medicine” AND rehabilitation and (*ii*) “implementation science” OR “implementation research” OR “implementation intervention” OR “implementation and dissemination” OR “implementation strategy” OR “implementation strategies” OR “implementation trials” AND rehabilitation.

### Selecting studies

The identified citations were imported into Zotero, and duplicates were removed. The selection process proceeded in stages. In the first stage, studies were screened by title and abstract, and those mentioning rehabilitation and implementation research (implementation research or implementation science) or knowledge translation were included. In the second stage, full-text articles were obtained and assessed against the inclusion criteria. The initial screening was conducted by 1 researcher (HH), while the full-text review was performed by 2 researchers (HH, MK). If consensus could not be reached during the second stage, a third researcher (RS-J) was consulted to make the final inclusion decision.

### Extracting data

Extraction was completed by 2 authors (HH, MK), and any discrepancies were resolved by the third author (RS-J) to reach consensus. Data extraction was conducted concurrently using ATLAS.ti (https://atlasti.com/) qualitative analysis software and Microsoft Excel (Microsoft Corp, Redmon, WA, USA) for data organization.

*Category 1.* The following characteristics of the articles were extracted: country, publication year, focus of the publication, study design, target population, study setting, and informants. The country was extracted according to the first author of the article. Publication year was the year in which the research article was first published.

*Category 2.* TMFs and text fragments describing the study purpose were extracted to explore which TMFs were used in the studies and the relationship between the TMFs used and the purpose of the study.

### Data analysis and synthesis

*Category 1.* The focus of the publication, target population, and study settings were grouped inductively. Data were collected from a range of sources across the studies. The informants – such as service providers, various professionals, and clients – were categorized as follows: (*i*) a single professional group (e.g., physiotherapists, speech therapists, occupational therapists, nurses); (*ii*) interprofessional groups consisting of different healthcare professionals; (*iii*) multi-perspective groups consisting of both healthcare professionals and clients; and (*iv*) clients only. In addition, we specified whether any of the professionals held leadership positions. The study design was coded into one of the following categories: quantitative research, qualitative research, case study, or mixed-methods research. These characteristics of studies were analysed using descriptive statistics.

*Category 2.* We conducted a deductive analysis to examine which TMFs were applied in the studies and how they aligned with the study purpose. We applied the 5 categories of theoretical approaches in implementation science introduced by Nilsen (15, 23): process models, determinant frameworks, classic theories, implementation theories, and evaluation frameworks.

Process models describe the stages involved in translating research into practice. Furthermore, some process models also provide practical guidance for planning and executing implementation efforts and/or selecting strategies to facilitate implementation.

Determinant frameworks, classic theories, and implementation theories aim to understand or explain the determinants of implementation outcomes. Moreover, determinant frameworks identify factors that may hinder or facilitate implementation and its outcomes, whereas both classic theories and implementation theories offer explanations of the aspects of implementation. However, the 2 theories differ in origin: classic theories are derived from other fields, e.g., psychology or sociology, and implementation theories are developed within implementation science, either from scratch or by adapting existing theories and concepts.

Finally, evaluation frameworks specify the components of implementation that can be assessed to determine the success of implementation.

To classify the study purposes, we applied a categorization proposed by Wang et al. (24), which builds on a framework developed by Birken et al. (17). More specifically, we applied 6 of the 9 research purposes identified by Wang et al. (24). During the iterative categorization process, 8 supplementary study purpose categories were added (Table I). During the categorization process, we systematically revisited the articles to verify whether the stated purposes were also reflected in the results section. Any discrepancies were resolved through consensus-seeking discussions between 2 authors (HH, MK). The third author (RS-J) was consulted when needed. Descriptive statistical methods were used to analyse the purposes of the studies and the TMFs used.

**Table I:** Categorization of the purposes of the studies

Categories of purposes proposed by Wang et al. (2023):
Evaluation of process
Guide design or selection of IS strategies
Guide implementation planning
Identify barriers and facilitators
Specify relationship between constructs or mechanism
Specify or describe process of implementation
Supplementary categories of purposes:
Describe current practice
Development of IS strategies
Development or adaptation of intervention
Development or adaptation of recommendations or pathways or guidelines
Evaluation of intervention outcomes
Identify core components
Identify IS strategies
Evaluation of implementation outcomes

IS: Implementation science.

The full data extraction template can be found in Appendix S2. The analysis was further conducted using the co-occurrence tool in ATLAS.ti. The analyses focused on the alignment between the classified purposes of the studies and the TMFs classified according to Nilsen’s categories (15, 23). Studies that employed a single TMF (n = 93) were included in the analysis. The remaining 28 studies, which employed more than 1 TMF, were analysed narratively.

## RESULTS

**Fig. 1:**
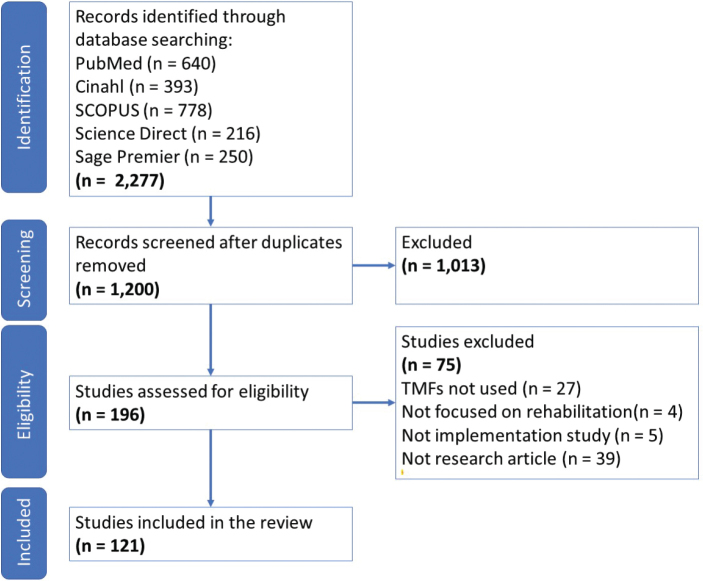
PRISMA flowchart of the literature search and publication selection.

A total of 2,277 citations were identified, and 121 articles were eligible for inclusion. This process and the reasons for exclusion are illustrated in Fig. 1. References of the included studies are provided in Appendix S3.

### General study characteristics

The general study characteristics are presented in Table II and Appendix S2.

**Table II:** Characteristics of the studies

	*n*	%
Country		
USA	35	29
Canada	32	26
Australia	24	20
Europe, including	24	20
Austria	1	1
Belgium	1	1
Denmark	2	2
France	1	1
Ireland	1	1
Netherlands	4	3
Norway	3	2
Sweden	3	2
UK	8	7
Saudi Arabia	2	2
China	1	1
New Zealand	1	1
South Africa	1	1
South Korea	1	1
Total	121	101
Publication year (version of record):		
2010	1	1
2011	0	0
2012	0	0
2013	2	2
2014	3	2
2015	8	7
2016	6	5
2017	4	3
2018	6	5
2019	11	9
2020	9	7
2021	23	19
2022	24	20
2023	18	15
2024	6	5
Total	121	100
Focus of journal:		
Rehabilitation	34	28
Profession-specific	28	23
Client group	18	15
Healthcare (broad)	18	15
Broad scope	16	13
Implementation science	5	4
Methodology	2	2
Total	121	100
Study design:		
Qualitative	60	50
Mixed-methods	39	32
Quantitative	18	15
Case report	4	3
Total	121	100
Target population:		
Neurological condition	44	36
Stroke	31	26
Spinal cord injury	6	5
Brain injury	4	3
Movement disorder, including Parkinson’s disease	3	2
Several	19	16
Paediatric	16	13
Aphasia	10	8
Cancer	5	4
Cardiovascular	5	4
Psychiatric	4	3
Geriatric	3	2
Other	14	12
Not reported	1	1
Total	121	100
Study settings:		
Multiple	37	31
Inpatient	34	28
Outpatient	17	14
Community	11	9
Regional/government	10	8
Home-based	6	5
Not reported	5	4
Research	1	1
Total	121	100
Informants:		
Interprofessional	47	39
Multi-perspective	32	26
Single professional group	31	26
Other	7	6
Clients	4	3
Total	121	100

Geographically, more than half of the studies were conducted in North America (55%), with 32 studies (26%) in Canada and 35 studies (29%) in the United States. In Australia, 24 studies (20%) were conducted. In Europe, 24 studies (20%) were conducted. Fewer studies originated from other regions: 2 studies from Saudi Arabia (2%) and 1 study (1%) each from China, New Zealand, South Africa, and South Korea.

Over the past decade, the number of rehabilitation studies employing TMFs has grown substantially. This upward trend is most pronounced from 2021 onwards: 9 studies were published in 2020, increasing to 23 in 2021 and 24 in 2022. The focus of the journals in which the studies were published was most often rehabilitation (*n* = 34; 28%), for example Disability and Rehabilitation, and profession-specific topics (*n* = 28; 23%), such as Physical & Occupational Therapy in Pediatrics. Additionally, studies appeared in journals targeting specific client groups (*n* = 18; 15%), for example International Psychogeriatrics, as well as journals broadly addressing healthcare (*n* = 18; 15%), such as BMC Health Services Research, and those with a broad scope (*n* = 16; 13%), such as PLOS ONE. Fewer studies were published in journals dedicated to implementation science (*n* = 5; 4%) or in methodology-focused journals (*n* = 2; 2%).

Of the included studies, half were qualitative (*n* = 60; 50%) and approximately one-third employed mixed-methods (*n* = 39; 32%). In addition, 18 studies (15%) were quantitative and 4 were case reports (3%). The target populations varied across studies. Most commonly, the focus was on the rehabilitation of individuals with neurological conditions: stroke in 31 studies (26%), spinal cord injury in 6 studies (5%), brain injury in 4 studies (3%), and movement disorders, including Parkinson’s disease, in 3 studies (2%). Furthermore, 16 studies (13%) targeted paediatric rehabilitation, and 10 studies (8%) focused on individuals with aphasia. Multiple target groups were included in 19 studies (16%), and 14 studies (12%) addressed other populations, such as people with chronic pain, osteoporosis, or amputations.

The study (and/or intervention) settings varied considerably: 34 (28%) were conducted in inpatient settings, 17 (14%) in outpatient settings, 11 (9%) in community settings, and 6 (5%) in home-based environments. In addition, 10 studies (8%) were carried out at the regional or governmental level. Most commonly, the studies involved multiple settings (n = 37; 31%). One study (1%) was conducted in a research setting, and for 5 studies (4%), the setting could not be identified.

Most commonly, data were collected from healthcare professionals, either interprofessionally (*n* = 47; 39%) or from a single professional group (*n* = 31; 26%). Data were also frequently collected multi-perspectively, including both professionals and clients (*n* = 32; 26%). Across these 3 categories involving data collection from professionals – interprofessional, multi- perspective, and single professional group – 26 studies (21%) included participants in leadership positions. Data were collected from clients, typically as part of a multi-perspective approach, but only rarely solely from rehabilitation clients (*n* = 4; 3%). In total, data from clients were collected in 36 studies (29%). In the remaining studies (*n* = 7; 6%), information was obtained from other sources, such as clinical research coordinators, documentation, or records.

### TMFs used in the studies

TMFs representing all 5 categories of the theoretical approaches outlined by Nilsen (15) were present in the studies (Table III; Appendix S2). TMFs within the determinant frameworks category were applied most frequently, appearing in 66 studies, followed by TMFs in the process models category, which appeared in 46 studies. TMFs in the implementation theories category were identified in 17 studies, while those in the evaluation frameworks category were applied in 14 studies. Finally, TMFs in the classic theories category were used in 3 studies, making it the least employed category. It should be noted that some studies applied more than 1 TMF within the same category.

**Table III:** TMFs reported in the included studies (*n* = 121)

TMF	*N*
Process models	48
Knowledge-to-Action (KTA)	33
Intervention Mapping (IM)	3
CAN-IMPLEMENT	2
Plan-Do-Study-Act (PDSA)	2
Adopt-Contextualize-Adapt (ACA)	1
Collaborative Intervention Planning Framework (CIPF)	1
French’s approach	1
Quality Implementation Framework (QIF)	1
Integrated Knowledge Translation (IKT)	1
Ottawa Model of Research Use	1
Replicating Effective Programmes framework (REP)	1
Stetler Model of Research Utilization	1
Determinant frameworks	76
Consolidated Framework for Implementation Research (CFIR)	43
Theoretical Domains Framework (TDF)	16
Expert Recommendations for Implementation Change (ERIC)	5
Integrated Promoting Action on Research Implementation in Health Services (iPARiHS)	5
Active Implementation Frameworks (AIF)	2
Grol and Wensing’s model	2
Exploration, Preparation, Implementation and Sustainment (EPIS)	1
Clinical Practice Guidelines Framework for Improvement	1
State Health Agency Yardstick (SHAY)	1
Implementation theories	18
Behaviour Change Wheel (BCW)	7
Behaviour Change Taxonomy	3
Capability, Opportunity, Motivation and Behaviour (COM-B)	3
Normalization Process Theory (NPT)	3
Implementation Research Logic Model	1
Target-Action-Context-Time-Actor (TACT-A)	1
Evaluation frameworks	14
Proctor’s taxonomy of implementation outcomes	5
Reach, Effectiveness, Adoption, Implementation, and Maintenance (RE-AIM)	5
Recommendations for the development, implementation, evaluation, and reporting of online KT resources	1
Nonadaptation, Abandonment, and Challenges to the Scale-up, Spread and Sustainability of Health and Care Technologies (NASSS) framework	1
Social Practice Theory	1
Theoretical Framework of Acceptability (TFA)	1
Classic theories	3
Theory of Diffusion of Innovation (DOI)	3

A total of 34 different TMFs were used in the included studies. Altogether 93 studies (77%) applied a single TMF, 22 studies (18%) applied 2 TMFs, 3 studies (3%) applied 3 TMFs, 2 studies (1%) applied 4 TMFs, and 1 study (1%) applied 5 TMFs, indicating considerable variation in the number of frameworks utilised across studies. The most frequently applied TMF was the Consolidated Framework for Implementation Research (CFIR) in 43 articles (36%), followed by the Knowledge-to-Action Framework (KTA) in 33 articles (27%), and the Theoretical Domains Framework (TDF) in 15 articles (12%). Nearly half of the TMFs (*n* = 16; 48%) were used in only 1 study.

### Relation between study purposes and used TMFs

A total of 242 study purposes were identified (see Appendix S2). The purposes of the studies most frequently fell into the “Identifying barriers and facilitators” category (*n* = 66; 27%) and the “Evaluating implementation outcomes” category (*n* = 43; 18%). In contrast, the least common purposes were the “Identify core components” category (*n* = 1; less than 1%) and the “Guide the design and selection of implementation strategies” category (*n* = 2; 1%) (Table IV).

**Table IV:** Purposes of the studies

Purpose	*n* (%)
Identify barriers and facilitators	66 (27)
Evaluation of implementation outcomes	43 (18)
Specify or describe process of implementation	24 (10)
Evaluation of process	18 (7)
Describe current practice	16 (7)
Identify IS strategies	16 (7)
Guide implementation planning	15 (6)
Development or adaptation of intervention	13 (5)
Development of IS strategies	9 (4)
Evaluation of intervention outcomes	9 (4)
Specify relationship between constructs or mechanism	7 (3)
Development or adaptation of recommendations or pathways or guidelines	3 (1)
Guide design or selection of IS strategies	2 (1)
Identify core components	1 (0)
Total number of purposes in the studies	242 (100)

IS: implementation science.

**Fig. 2:**
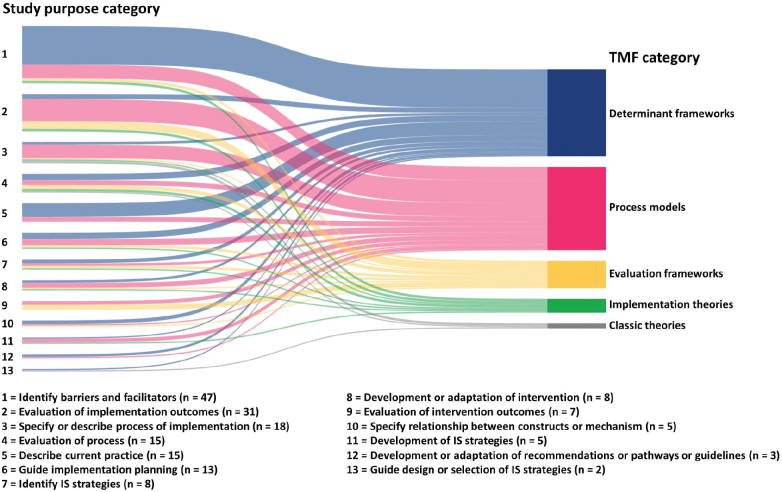
Alignment between study purpose and TMF categories in studies (*n* = 93) that applied 1 TMF.

Fig. 2 illustrates the number of studies in each of the 143 study-purpose categories listed in Table I, the number of applied TMFs across the 5 categories proposed by Nilsen (15, 23), and the alignment between study purpose and TMF category in studies that applied a single TMF (*n* = 93). In these studies, we identified a total of 177 study purposes. Among these studies, the most frequently applied TMFs were in the determinant frameworks category (*n* = 43), followed by TMFs in the process models category (*n* = 34) and the evaluation frameworks category (*n* = 9). TMFs in the implementation theories and classic theories categories (*n* = 5; *n* = 2, respectively) were applied in only a small number of studies.

The most common study-purpose category was the “Identify barriers and facilitators” (*n* = 47). In these studies, TMFs within the determinant frameworks category were used in three-quarters of cases (*n* = 32), followed by TMFs in the process models category (*n* = 11).

The “Evaluation of implementation outcomes” (*n* = 31) was the second most common study-purpose category. In this category, TMFs in the process models category (*n* = 19) were applied in over half of the studies. TMFs in the evaluation frameworks (*n* = 6) and determinant frameworks (*n* = 4) categories were applied at nearly equal frequency in these studies.

The third most common study-purpose category was the “Specify or describe process of implementation” (*n* = 18). In this category, TMFs in the process models category (*n* = 12) were applied most commonly. TMFs in both classic theories and determinant frameworks categories were used in 2 studies, while TMFs in evaluation frameworks and implementation theories categories were each employed in 1 study.

When the study purpose fell under the “Evaluation of process” category (*n* = 15), TMF use varied across all 5 of Nilsen’s categories. TMFs within determinant frameworks were applied in 5 studies, those within process models in 4, evaluation frameworks in 3, implementation theories in 2, and classic theories in 1.

In studies with the purpose of “Describing current practice” (*n* = 15), only TMFs within determinant frameworks (*n* = 11) and process models (*n* = 4) were used.

Among studies in the category “Guiding implementation planning” (*n* = 13), TMFs within determinant frameworks (*n* = 5) and process models (*n* = 5) were utilized with the same frequency. The use of TMFs within implementation theories (*n* = 1) and evaluation frameworks (*n* = 2) was limited.

A total of 28 studies employed more than 1 TMF. Most of them of them used 2 TMFs (*n* = 22). The most common combination was to use 2 TMFs within determinant frameworks (*n* = 8), for example CFIR and TDF or CFIR and Expert Recommendations for Implementation Change (ERIC). The second most common combination was to use a TMF within determinant frameworks and a TMF within implementation theories (*n* = 6), for example TDF and Capability, Opportunity, Motivation and Behaviour (COM-B). The third most common combination was to combine TMFs within determinant frameworks and process models (*n* = 4), for example CFIR and KTA. A combination of TMFs within implementation theories and process models was employed in 2 studies; in addition evaluation frameworks and process models, and determinant and classic theories were each employed in 1 study. The remaining 6 studies applied 3–5 TMFs in various combinations (see Appendix S2).

## DISCUSSION

This scoping review explored rehabilitation studies that applied implementation science TMFs, focusing on study characteristics and the alignment between TMF use and study purposes. The analysis of 121 articles revealed a growing trend in applying TMFs within rehabilitation research, although their overall prevalence remains modest compared with the broader rehabilitation literature. The studies demonstrated considerable diversity in client groups, settings, and perspectives, most often adopting interprofessional or multi- perspective approaches. Methodologically, qualitative designs predominated, with one-third of the studies using mixed-methods, reflecting the complexity of implementation processes. A total of 34 different TMFs were identified, covering all 5 categories proposed by Nilsen (15, 23). The analysis showed that study purposes and TMF categories sometimes aligned. For instance, determinant frameworks were often used to identify barriers and facilitators. However, there were also cases where the connection was unclear, raising questions about the appropriateness of the chosen TMF.

### Characteristics of rehabilitation implementation studies that applied TMFs

Although the number of rehabilitation studies using TMFs is increasing, it remains small relative to the overall research volume. This mirrors broader trends in health research, where implementation science has grown but still represents a modest proportion (25). Kinney et al. (13) found that only 38% of studies on barriers and facilitators in occupational and physical therapy explicitly used TMFs, underscoring the need for more systematic application. Expanding implementation research is essential to accelerate the translation of evidence-based interventions into practice, as research evidence can take up to 17 years to reach routine care (26). Incorporating implementation science approaches into randomized controlled trials is one strategy to narrow this gap (27).

The articles included in this review reflect the diversity of rehabilitation, which is a key strength of implementation research in this field. Another strength lies in the inclusion of diverse informants – such as service providers, various professionals, and clients – and the use of multiple methods. Although the client groups were varied, neurological conditions and paediatric populations were the most commonly represented groups. Notably, 29% of the studies included clients as informants. However, the client perspective could be emphasized more strongly in implementation research, given rehabilitation’s focus on client needs and person-centredness. Unlike many medical procedures where patients are passive recipients, rehabilitation positions the client as an active participant (28). One factor limiting this perspective may be that not all TMFs explicitly incorporate client-related constructs (cf. 23). This finding also highlights an important consideration for future research: TMFs selected for rehabilitation implementation studies should be assessed in terms of their ability to capture client-related constructs, such as goals, preferences, and engagement. Where suitable TMFs are not available, it may be warranted to adapt or extend existing frameworks to incorporate these constructs.

This review suggests that implementation research should extend beyond direct practice to include managerial, organizational, and policy levels. Managers appeared as informants in only 21% of the studies, despite their critical role in sustaining interventions (29). Policy considerations should be integrated during research rather than addressed only at implementation. TMFs can frame policy influence, promote evidence-based policies, and support de-implementation of ineffective practices (30).

### Alignment between TMF use and study purposes

This review suggests that the use of determinant frameworks most often aligns with the purposes of the studies. According to Nilsen (15), determinant frameworks are intended to explain which different-level determinants affect the implementation outcomes and act as barriers or facilitators. In this study, when the purpose of a study was to identify barriers and facilitators, determinant frameworks were used in three-quarters of the cases, which aligns with Nilsen’s definition. Another substantial group where determinant frameworks were commonly used consisted of studies aimed at describing current practice. This may be due to the ability of determinant frameworks to capture context-specific factors that support describing the current practice (15). In addition, in studies focused on evaluating implementation outcomes, determinant frameworks and evaluation frameworks were applied with similar frequency. In light of Nilsen’s categories, both determinant and evaluation frameworks can be regarded as an appropriate option. While evaluation frameworks are designed to provide structure for assessing implementation (15), determinant frameworks primarily describe factors influencing implementation and outcomes, which may vary depending on the implementation object (15).

Process models are another major TMF category in rehabilitation implementation research; however, their use did not always align with the study purpose. For instance, process models were the second most frequently applied TMFs when the aim was to identify barriers and facilitators, even though Nilsen (15) argues that determinant frameworks are better suited for explaining factors influencing implementation, while process models are intended to describe the stages of the research-to-practice process. Similarly, process models were chosen in more than half of the studies focusing on evaluating implementation outcomes, despite their primary focus not being outcome assessment – although outcomes can be considered at different phases of implementation. Conversely, process models were appropriately used in studies aiming to specify or to describe the implementation process.

In studies where the purpose was the evaluation of process, TMFs were applied with considerable diversity across the 5 Nilsen categories. According to Nilsen’s (23) definition, process models incorporate a temporal sequence of implementation activities, which can guide the evaluation of the process, whereas determinant frameworks focus on influencing factors and do not explicitly adopt a process-oriented perspective.

The results of this study showed that the selection of TMFs did not always consistently align with the study purpose. However, it should be noted that many factors within the study purpose affect the selection of TMFs. Fontaine et al. (31) conclude that TMFs are often selected based on how familiar researchers are with them, rather than how suitable they are for the purpose of the study. In this review, the most frequently applied TMFs were KTA, TDF, and CFIR. The same TMFs are among the most commonly identified TMFs in other disciplines and sources as well (32, 33). However, it is important to acknowledge that rehabilitation research also demonstrates small-scale use of a wide range of other TMFs. As identified in this review, rehabilitation implementation studies have multiple purposes, and often a single framework cannot fully capture the complexity of the phenomenon under investigation. Therefore, it is common, and even recommended, to employ multiple frameworks within 1 study (10, 32, 34). It is crucial to leverage the available tools and resources designed to guide the selection of TMFs, as they enable meaningful application and help address broader considerations, such as equity and cultural safety (31, 35).

### Strengths and limitations

A key strength of this review is that a predefined and systematic search process was followed, with an information specialist involved to ensure comprehensive coverage of relevant articles. The selection of primary studies and data extraction were conducted by the research team according to agreed procedures, with discussions held to resolve differing interpretations. Data analysis was carried out using jointly established methods, and any challenges were addressed collaboratively. Additionally, ATLAS.ti software was employed to facilitate a structured and reproducible data extraction process. The research team included authors with expertise in scoping review methodology, implementation science, and rehabilitation.

Despite the systematic approach, some limitations remain. The implementation of different stages of the study may have introduced a risk of bias. During the selection process, drawing from a large pool of studies, some relevant articles may have been overlooked. Both key concepts – rehabilitation and implementation research – are inherently ambiguous, which may have contributed to the omission of essential articles. Furthermore, during data extraction and synthesis, some relevant information may not have been fully captured. These strengths and limitations should be considered when interpreting the findings of this review.

### Implications for future research and practice

The use of theories, models, and frameworks (TMFs) in implementation science has increased, but their application is often superficial and not always aligned with study objectives (25). This superficiality may result from uninformed or convenience-based choices rather than deliberate alignment with research aims. In the future, whether the selected TMFs fit the study’s purpose and how they affect the research design, methods, and interpretation of results should be carefully assessed.

In rehabilitation, there is a pressing need for implementation research that applies TMFs for a variety of purposes. While TMFs have been widely used, for example, to identify barriers and facilitators or to evaluate implementation outcomes, research should increasingly target areas that remain underexplored. Specifically, future studies should focus on identifying the mechanisms through which implementation occurs and how effective interventions achieve their outcomes, as well as determining the core components of interventions, programmes, or services that are essential for success.

The ultimate goal of implementation science TMFs and related research is to ensure that knowledge generated in scientific studies is applied in real-world practice. Bridging the knowledge–practice gap requires translating research findings into user-friendly tools that practitioners can readily use (36). At their best, TMFs provide a common language for collaboration among researchers, practitioners, policy-makers, and rehabilitation clients. They can connect research and practice and be applied not only in research but also in development projects. To achieve this, both rigorous scientific publications and accessible, practice-oriented materials are needed to support effective knowledge translation.

### Conclusion

This study advances our understanding of how TMFs are used in rehabilitation research and how study purposes and TMFs align. It highlights the importance of selecting TMFs that align with the purposes of an implementation project to facilitate the meaningful use of TMFs.

The use of implementation science in rehabilitation remains limited despite its growing importance. To close the research–practice gap, future studies should not only focus on initial uptake but also on sustaining evidence-based practices over time. Applying TMFs is essential for understanding the mechanisms of sustainment and developing strategies that ensure long-term integration into real-world settings.

## Supplementary Material


