# Supplementary material for: THEORIES, MODELS, AND FRAMEWORKS IN IMPLEMENTATION SCIENCE IN THE CONTEXT OF REHABILITATION RESEARCH: A SCOPING REVIEW
Source: J Rehabil Med. 2026 Jul 17;58:46016. doi: 10.2340/jrm.v58.46016 (PMC13389747; doi:10.2340/jrm.v58.46016)
Supplement: Supplementary file 1 [file JRM-58-46016-s1.pdf]

Supplementary material has been published as submitted. It has not been copyedited, or typeset by Journal of Rehabilitation Medicine

Appendix S1. Preferred Reporting Items for Systematic reviews and Meta-Analyses extension for Scoping Reviews (PRISMA-ScR) Checklist

SECTION	ITEM	PRISMA-ScR CHECKLIST ITEM	REPORTED ON PAGE #
**TITLE**			
Title	1	Identify the report as a scoping review.	1
**ABSTRACT**			
Structured summary	2	Provide a structured summary that includes (as applicable): background, objectives, eligibility criteria, sources of evidence, charting methods, results, and conclusions that relate to the review questions and objectives.	2
**INTRODUCTION**			
Rationale	3	Describe the rationale for the review in the context of what is already known. Explain why the review questions/objectives lend themselves to a scoping review approach.	3-4
Objectives	4	Provide an explicit statement of the questions and objectives being addressed with reference to their key elements (e.g., population or participants, concepts, and context) or other relevant key elements used to conceptualize the review questions and/or objectives.	5-6
**METHODS**			
Protocol and registration	5	Indicate whether a review protocol exists; state if and where it can be accessed (e.g., a Web address); and if available, provide registration information, including the registration number.	5
Eligibility criteria	6	Specify characteristics of the sources of evidence used as eligibility criteria (e.g., years considered, language, and publication status), and provide a rationale.	6
Information sources*	7	Describe all information sources in the search (e.g., databases with dates of coverage and contact with authors to identify additional sources), as well as the date the most recent search was executed.	6
Search	8	Present the full electronic search strategy for at least 1 database, including any limits used, such that it could be repeated.	6
Selection of sources of evidence†	9	State the process for selecting sources of evidence (i.e., screening and eligibility) included in the scoping review.	6
Data charting process‡	10	Describe the methods of charting data from the included sources of evidence (e.g., calibrated forms or forms that have been tested by the team before their use, and whether data charting was done independently or in duplicate) and any processes for obtaining and confirming data from investigators.	6-7
Data items	11	List and define all variables for which data were sought and any assumptions and simplifications made.	6-7
Critical appraisal of individual sources of evidence§	12	If done, provide a rationale for conducting a critical appraisal of included sources of evidence; describe the methods used and how this information was used in any data synthesis (if appropriate).	N/A
Synthesis of results	13	Describe the methods of handling and summarizing the data that were charted.	7-8
**RESULTS**			
Selection of sources of evidence	14	Give numbers of sources of evidence screened, assessed for eligibility, and included in the review, with reasons for exclusions at each stage, ideally using a flow diagram.	8
Characteristics of sources of evidence	15	For each source of evidence, present characteristics for which data were charted and provide the citations.	8
Critical appraisal within sources of evidence	16	If done, present data on critical appraisal of included sources of evidence (see item 12).	N/A

SECTION	ITEM	PRISMA-ScR CHECKLIST ITEM	REPORTED ON PAGE #
Results of individual sources of evidence	17	For each included source of evidence, present the relevant data that were charted that relate to the review questions and objectives.	8-12
Synthesis of results	18	Summarize and/or present the charting results as they relate to the review questions and objectives.	8-12
**DISCUSSION**			
Summary of evidence	19	Summarize the main results (including an overview of concepts, themes, and types of evidence available), link to the review questions and objectives, and consider the relevance to key groups.	12-14
Limitations	20	Discuss the limitations of the scoping review process.	15
Conclusions	21	Provide a general interpretation of the results with respect to the review questions and objectives, as well as potential implications and/or next steps.	16
**FUNDING**			
Funding	22	Describe sources of funding for the included sources of evidence, as well as sources of funding for the scoping review. Describe the role of the funders of the scoping review.	16

From: Tricco AC, Lillie E, Zarin W, O'Brien KK, Colquhoun H, Levac D, et al. PRISMA Extension for Scoping Reviews (PRISMAScR): Checklist and Explanation. Ann Intern Med. 2018;169:467–473. doi: 10.7326/M18-0850.

Appendix SII. The general study characteristics, TMFs used in the studies and study purposes in included studies (n = 121)

**Study (AU, year, country)**	**Study design**	**Informants**	**Target population**	**Study setting**	**TMFs reported in the included studies**	**Categories of theoretical approaches outlined by Nilsen (2015; 2024)**	**Study purpose**
**Adsul et al. 2022, USA**	Mixed methods	Multiperspective (including participants in leadership positions)	Cancer	Multiple	Exploration, Preparation, Implementation and Sustainment (EPIS)	Determinant framework	Guide design or selection of IS strategies<br>Identify barriers and facilitators<br>Specify or describe process of implementation
**Ahmed et al. 2021, Canada**	Mixed methods	Multiperspective (including participants in leadership positions)	Other (chronic pain)	Multiple	Consolidated Framework for Implementation Research (CFIR)<br>Theoretical Domains Framework (TDF)	Determinant framework	Guide implementation planning<br>Identify barriers and facilitators<br>Identify IS strategies
**Alatawi et al. 2022, Saudi Arabia**	Qualitative	One Profession (including participants in leadership positions)	Neurological (stroke)	Outpatient	Integrated Promoting Action on Research Implementation in Health Services (iPARiHS)	Determinant framework	Development or adaptation of recommendations or pathways or guidelines
**Alatawi. 2019, Saudi Arabia**	Mixed methods	One Profession	Neurological (stroke)	Regional /government	Knowledge-to-Action (KTA)	Process model	Guide implementation planning<br>Identify barriers and facilitators
**Allen et al. 2020, Canada**	Qualitative	Interprofessional	Neurological (stroke)	Inpatient	Integrated Promoting Action on Research Implementation in Health Services (iPARiHS)	Determinant framework	Development or adaptation of recommendations or pathways or guidelines<br>Guide implementation planning
**Arnold et al. 2020, Australia**	Quantitative	One Profession	Aphasia	Multiple	Theoretical Domains Framework (TDF)	Determinant framework	Describe current practice<br>Identify barriers and facilitators

**Auld & Johnston 2019, Australia**	Mixed methods	Interprofessional	Paediatric	Not reported	Knowledge-to-Action (KTA)	Process model	Evaluation of implementation outcomes<br>Guide implementation planning<br>Identify IS strategies<br>Identify barriers and facilitators
**Bird et al. 2019, Canada**	Qualitative	Multiperspective (including participants in leadership positions)	Neurological (stroke)	Community	Normalization Process Theory (NPT)	Implementation theory	Development or adaptation of intervention<br>Evaluation of process<br>evaluation of implementation outcomes
**Bond et al. 2021, USA**	Qualitative	Multiperspective (including participants in leadership positions)	Other (employment)	Regional /government	State Health Agency Yardstick (SHAY)	Determinant framework	Identify barriers and facilitators<br>Identify IS strategies
**Bozec et al. 2021, USA**	Mixed methods	One Profession	Several	Inpatient	Consolidated Framework for Implementation Research (CFIR)<br>Theoretical Domains Framework (TDF)	Determinant framework	Development or adaptation of intervention<br>Identify barriers and facilitators
**Brouns et al. 2018, Netherlands**	Qualitative	Interprofessional (including participants in leadership positions)	Neurological (stroke)	Outpatient	Grol's and Wensing's model	Determinant framework	Identify barriers and facilitators
**Cahill et al. 2021, Australia**	Qualitative	Interprofessional	Neurological (stroke)	Not reported	Normalization Process Theory (NPT)<br>Theoretical Domains Framework (TDF)	Implementation theory<br>Determinant framework	Identify barriers and facilitators
**Camden et al. 2015, Canada**	Quantitative	One Profession	Paediatric	Multiple	Knowledge-to-Action (KTA)	Process model	Evaluation of implementation outcomes<br>Evaluation of process

**Celian et al. 2021, USA**	Qualitative	Interprofessional	Several	Inpatient	Consolidated Framework for Implementation Research (CFIR)	Determinant framework	Identify barriers and facilitators
**Clare et al. 2023, UK**	Mixed methods	Multiperspective	Other (dementia)	Community	Knowledge-to-Action (KTA)<br>Ottawa Model of Research Use<br>Integrated Promoting Action on Research Implementation in Health Services (iPARiHS)<br>Stetler Model of Research Utilization<br>Proctor's taxonomy of implementation outcomes	Process model<br>Determinant framework<br>Evaluation framework	Evaluation of implementation outcomes<br>Evaluation of intervention outcomes<br>Evaluation of process<br>Identify barriers and facilitators
**Connell et al. 2021, New Zealand**	Qualitative	Interprofessional	Neurological (stroke)	Regional /government	Normalization Process Theory (NPT)<br>Consolidated Framework for Implementation Research (CFIR)	Implementation theory<br>Determinant framework	Identify barriers and facilitators<br>Identify IS strategies
**Connell et al. 2014, UK**	Quantitative	Interprofessional	Neurological (stroke)	Multiple	Consolidated Framework for Implementation Research (CFIR)	Determinant framework	Describe current practice
**Connell et al. 2016a, UK**	Qualitative	Multiperspective	Neurological (stroke)	Inpatient	Behaviour Change wheel (BCW)	Implementation theory	Evaluation of process<br>Evaluation of implementation outcomes
**Connell et al. 2016b, UK**	Qualitative	Interprofessional (including participants in leadership positions)	Neurological (stroke)	Inpatient	Behaviour Change wheel (BCW)<br>Theoretical Domains Framework (TDF)	Implementation theory<br>Determinant framework	Specify relationship between constructs or mechanism

**Corbin et al. 2023, France**	Qualitative	Interprofessional	Neurological (stroke)	Inpatient	Consolidated Framework for Implementation Research (CFIR)	Determinant framework	Describe current practice<br>Identify barriers and facilitators
**Cox et al. 2021, Australia**	Quantitative	One Profession	Several	Community	Capability, Opportunity, Motivation and Behaviour (COM-B)<br>Theoretical Domains Framework (TDF)	Implementation theory<br>Determinant framework	Development of IS strategies<br>Evaluation of implementation outcomes<br>Identify barriers and facilitators
**Cunningham et al 2018, Canada**	Quantitative	One Profession	Paediatric	Community	Theory of Diffusion of Innovation (DOI)	Classic theory	Specify or describe process of implementation
**Cunningham et al. 2016, Canada**	Quantitative	One Profession	Paediatric	Community	Theory of Diffusion of Innovation (DOI)	Classic theory	Guide design or selection of IS strategies<br>Evaluation of process<br>Specify or describe process of implementation
**Daoust et al. 2021, Canada**	Qualitative	One Profession	Paediatric	Multiple	Consolidated Framework for Implementation Research (CFIR)<br>Knowledge-to-Action (KTA)	Determinant framework<br>Process model	Development or adaptation of intervention
**Demers et al. 2015, Canada**	Qualitative	Interprofessional	Several	Multiple	Knowledge-to-Action (KTA)	Process model	Identify barriers and facilitators
**Déry et al. 2021, Canada**	Mixed methods	Interprofessional (including participants in leadership positions)	Several	Multiple	Consolidated Framework for Implementation Research (CFIR)	Determinant framework	Identify barriers and facilitators
**D'Souza et al. 2022, Australia**	Qualitative	Interprofessional	Aphasia	Inpatient	Behaviour Change Taxonomy<br>Behaviour Change wheel (BCW)	Implementation theory<br>Process model	Evaluation of process<br>Evaluation of implementation outcomes<br>Identify barriers and facilitators

					Knowledge-to-Action (KTA)		
**Duran et al. 2023; USA**	Mixed methods	Interprofessional (including participants in leadership positions)	Cardiovascular	Outpatient	Consolidated Framework for Implementation Research (CFIR)<br>Theoretical Domains Framework (TDF)	Determinant framework	Development or adaptation of intervention<br>Identify barriers and facilitators<br>Spesify or describe process of implementation
**Eakin et al. 2015, USA**	Qualitative	Interprofessional	Several	Inpatient	Consolidated Framework for Implementation Research (CFIR)	Determinant framework	Evaluation of process<br>Evaluation of implementation outcomes<br>Identify barriers and facilitators<br>Identify IS strategies
**Fehlings et al. 2017, Canada**	Qualitative	Multiperspective (including participants in leadership positions)	Neurological (spinal cord injury)	Regional /government	Knowledge-to-Action (KTA)	Process model	Guide implementation planning
**Ferro & Quinn 2020, USA**	Mixed methods	One Profession	Paediatric	Community	Knowledge-to-Action (KTA)	Process model	Evaluation of implementation outcomes<br>Identify barriers and facilitators
**Frost et al. 2020, UK**	Mixed methods	Clients	Cardiovascular	Home-based	Social practice theory	Evaluation frameworks	Specify relationship between constructs or mechanism
**Gaid et al. 2023, Canada**	Qualitative	Interprofessional	Several	Multiple	Consolidated Framework for Implementation Research (CFIR)	Determinant framework	Identify barriers and facilitators
**Giurleo et al. 2022, UK**	Mixed methods	Multiperspective	Neurological (spinal cord injury)	Inpatient	Active Implementation Frameworks Plan-Do-Study-Act (PDSA)	Determinant framework<br>Process model	Evaluation of implementation outcomes<br>Identify core components<br>Specify or describe process of implementation

**Gorzelitz et al. 2022, USA**	Qualitative	Interprofessional	Cancer	Multiple	Consolidated Framework for Implementation Research (CFIR)<br>Expert Recommendations for Implementation Change (ERIC)	Determinant framework	Describe current practice<br>Identify barriers and facilitators<br>Identify IS strategies<br>Specify relationship between constructs or mechanism
**Grimmer et al. 2019, South Africa**	Mixed methods	Multiperspective (including participants in leadership positions)	Neurological (stroke)	Regional /government	Adopt-Contextualize-Adapt (ACA)	Process model	Specify or describe process of implementation
**Hadely et al. 2014, Australia**	Quantitative	One Profession	Neurological (stroke)	Multiple	Knowledge-to-Action (KTA)	Process model	Describe current practice<br>Identify barriers and facilitators<br>Identify IS strategies
**Heinemann et al. 2022, USA**	Qualitative	Other	Neurological (spinal cord injury)	Inpatient	CAN-IMPLMENTENT	Process model	Development or adaptation of intervention<br>Specify or describe process of implementation
**Hirschhorn et al. 2013, Australia**	Qualitative	Multiperspective	Other (incontinence)	Multiple	French's approach	Process model	Identify barriers and facilitators
**Holmlund et al. 2022, Sweden**	Qualitative	Multiperspective	Psychiatric	Regional /government	Consolidated Framework for Implementation Research (CFIR)	Determinant framework	Identify barriers and facilitators
**Hreha et al. 2022, USA**	Mixed methods	Interprofessional (including participants in leadership positions)	Neurological (stroke)	Inpatient	Consolidated Framework for Implementation Research (CFIR)<br>Knowledge-to-Action (KTA)	Determinant framework<br>Process model	Evaluation of implementation outcomes<br>Identify barriers and facilitators<br>Specify or describe process of implementation

**Imms et al. 2021, Australia**	Quantitative	Multiperspective	Paediatric	Outpatient	Consolidated Framework for Implementation Research (CFIR)<br>Theory of Diffusion of Innovation (DOI)	Determinant framework<br>Classic theory	Evaluation of implementation outcomes
**Inness et al. 2022, Canada**	Qualitative	Interprofessional (including participants in leadership positions)	Neurological (stroke)	Multiple	Consolidated Framework for Implementation Research (CFIR)<br>Theoretical Domains Framework (TDF)	Determinant framework	Identify barriers and facilitators<br>Development of IS strategies
**Jeong et al. 2016, Canada**	Quantitative	One Profession	Paediatric	Multiple	Knowledge-to-Action (KTA)	Process model	Describe current practice<br>Evaluation of implementation outcomes<br>Evaluation of process<br>Guide implementation planning<br>Identify barriers and facilitators<br>Specify or describe process of implementation
**Jervis Rademeyer et al. 2023, Canada**	Qualitative	Interprofessional	Neurological (spinal cord injury)	Multiple	Theoretical Domains Framework (TDF)	Determinant framework	Describe current practice
**Johanson et al. 2020, Sweden**	Qualitative	Interprofessional (including participants in leadership positions)	Psychiatric	Outpatient	Consolidated Framework for Implementation Research (CFIR)	Determinant framework	Evaluation of process<br>Evaluation of implementation outcomes<br>Identify barriers and facilitators
**Johnston et al. 2023, Australia**	Qualitative	Interprofessional	Neurological (brain injury)	Regional /government	Consolidated Framework for Implementation Research (CFIR)	Determinant framework	Describe current practice

**Jolliffe et al. 2024, Australia**	Qualitative	Interprofessional (including participants in leadership positions)	Several	Community	Behaviour Change wheel (BCW)<br>Theoretical Domains Framework (TDF)	Implementation theory<br>Determinant framework	Identify barriers and facilitatiors<br>Identify IS strategies
**Jones et al. 2021, Canada**	Qualitative	Interprofessional	Neurological (brain injury)	Multiple	Active Implementation Frameworks (AIF)	Determinant framework	Identify barriers and facilitatiors<br>Identify IS strategies
**Kang et al. 2023, USA**	Mixed methods	Multiperspective	Several	Community	Intervention Mapping (IM)<br>Proctor's taxonomy of implementation outcomes	Process model<br>Evaluation framework	Evaluation of implementation outcomes
**Kang & Foster. 2022, USA**	Qualitative	Multiperspective	Several	Community	Behaviour Change Taxonomy<br>Consolidated Framework for Implementation Research (CFIR)<br>Intervention Mapping (IM)<br>Proctor's taxonomy of implementation outcomes	Implementation theory<br>Determinant framework<br>Process model<br>Evaluation framework	Development of IS strategies<br>Specify or describe process of implementation
**Karlsson et al. 2023, Sweden**	Qualitative	Interprofessional (including participants in leadership positions)	Psychiatric	Regional /government	Consolidated Framework for Implementation Research (CFIR)	Determinant framework	Evaluation of process<br>Identify barriers and facilitators
**Kelly et al. 2022, UK**	Qualitative	Interprofessional	Paediatric	Outpatient	Plan-Do-Study-Act (PDSA)	Process model	Evaluation of implementation outcomes
**Kengne Talla et al. 2023, Canada**	Mixed methods	Other	Several	Research	Integrated Promoting Action on Research Implementation in Health Services (iPARiHS)	Determinant framework	Identify barriers and facilitators

**Kettlewell et al. 2022, UK**	Qualitative	Multiperspective	Other (traumatic injuries)	Multiple	Consolidated Framework for Implementation Research (CFIR)	Determinant framework	Identify barriers and facilitators
**Kim et al. 2021, South Korea**	Qualitative	Multiperspective	Neurological (stroke)	Inpatient	Knowledge-to-Action (KTA)	Process model	Development or adaptation of intervention
**Kingsnorth et al. 2020, Canada**	Qualitative	Interprofessional (including participants in leadership positions)	Paediatric	Inpatient	Knowledge-to-Action (KTA)	Process model	Development or adaptation of recommendations or pathways or guidelines<br>Evaluation of implementation outcomes<br>Specify or describe process of implementation
**Kristensen & Hounsgaard. 2013, Denmark**	Qualitative	Interprofessional	Several	Regional /government	Integrated Promoting Action on Research Implementation in Health Services (iPARiHS)	Determinant framework	Guide implementation planning<br>Identify barriers and facilitators
**Lamper et al. 2022, Netherlands**	Qualitative	Multiperspective	Other (chronic pain)	Multiple	Consolidated Framework for Implementation Research (CFIR)	Determinant framework	Evaluation of implementation outcomes<br>Identify barriers and facilitators
**Levy et al. 2022, Australia**	Mixed methods	Multiperspective	Neurological (stroke)	Inpatient	Proctor's taxonomy of implementation outcomes	Evaluation frameworks	Development or adaptation of intervention<br>Evaluation of implementation outcomes<br>Evaluation of intervention outcomes<br>Identify IS strategies

**Lundquist ym. 2021, Denmark**	Qualitative	Interprofessional	Neurological (stroke)	Inpatient	Consolidated Framework for Implementation Research (CFIR)	Determinant framework	Describe current practice<br>Guide implementation planning<br>Identify barriers and facilitators
**Lynch et al. 2022, Australia**	Mixed methods	Interprofessional	Neurological (stroke)	Regional /government	Consolidated Framework for Implementation Research (CFIR)	Determinant framework	Describe current practice<br>Identify barriers and facilitators
**Mayo et al. 2023, Canada**	Qualitative	Clients	Other (amputation)	Multiple	Consolidated Framework for Implementation Research (CFIR)	Determinant framework	Guide implementation planning
**Mc Sharry et al. 2016, Ireland**	Mixed methods	Interprofessional	Cardiovascular	Outpatient	Behaviour Change wheel (BCW)	Implementation theory	Guide implementation planning
**Mc Arthur et al. 2018, Canada**	Qualitative	Interprofessional	Other (osteoporosis)	Multiple	Behaviour Change wheel (BCW)	Implementation theory	Identify barriers and facilitators<br>Identify IS strategies<br>Specify or describe process of implementation
**Mc Ewen et al. 2019, Canada**	Quantitative	Interprofessional	Neurological (stroke)	Inpatient	Knowledge-to-Action (KTA)	Process model	Evaluation of implementation outcomes
**Miao et al. 2023, Australia**	Mixed methods	Multiperspective (including participants in leadership positions)	Neurological (brain injury)	Multiple	Nonadaptation, Abandonment, and Challenges to the Scale-up, Spread and Sustainability of Health and Care Technologies (NASSS) framework	Evaluation framework	Guide implementation planning<br>Identify IS strategies
**Moore et al. 2018, Norway**	Quantitative	Interprofessional	Several	Multiple	Knowledge-to-Action (KTA)	Process model	Describe current practice<br>Evaluation of implementation outcomes

**Moore et al. 2022, Norway**	Quantitative	One Profession	Neurological (stroke)	Inpatient	Knowledge-to-Action (KTA)	Process model	Evaluation of implementation outcomes
**Moore et al. 2021, Norway**	Mixed methods	One Profession	Neurological (stroke)	Inpatient	Consolidated Framework for Implementation Research (CFIR)<br>Knowledge-to-Action (KTA)	Determinant framework<br>Process model	Evaluation of process<br>Evaluation of implementation outcomes<br>Identify barriers and facilitators<br>Identify IS strategies<br>Development or adaptation intervention<br>Specify or describe process of implementation
**Munce et al. 2017, Canada**	Qualitative	Interprofessional (including participants in leadership positions)	Neurological (stroke)	Not reported	Clinical Practice Guidelines Framework for Improvement	Determinant framework	Identify barriers and facilitators
**Nitsch et al. 2021, USA**	Qualitative	Interprofessional	Neurological (spinal cord injury)	Inpatient	Knowledge-to-Action (KTA)<br>CAN-IMPLEMENT<br>Target-Action-Context-Time-Actor (TACT-A)	Process model<br>Implementation theory	Development or adaptation of intervention<br>Guide implementation planning<br>Identify barriers and facilitators
**Orava et al. 2019, Canada**	Mixed methods	Interprofessional	Paediatric	Outpatient	Knowledge-to-Action (KTA)	Process model	Evaluation of process<br>Evaluation of implementation outcomes<br>Development or adaptation of intervention<br>Identify barriers and facilitators
**Pastva et al. 2021, USA**	Mixed methods	Interprofessional	Neurological (stroke)	Multiple	Consolidated Framework for Implementation Research (CFIR)	Determinant framework	Describe current practice<br>Evaluation of process

**Patterson et al.<br>2024,<br>Australia**	Qualitative	Clients	Cardiovascular	Home-based	Capability, Opportunity, Motivation and Behaviour (COM-B)<br>Theoretical Domains Framework (TDF)	Implementation theory<br>Determinant framework	Identify barriers and facilitators
**Pepin et al.<br>2024,<br>USA**	Mixed methods	One Profession	Other<br>(low back pain)	Outpatient	Knowledge-to-Action (KTA)	Process model	Development of IS strategies<br>Evaluation of implementation outcomes<br>Specify or describe process of implementation
**Perez et al.<br>2017,<br>Canada**	Mixed methods	Multiperspective	Neurological (stroke)	Inpatient	Consolidated Framework for Implementation Research (CFIR)	Determinant framework	Evaluation of process
**Pittman et al.<br>2021,<br>USA**	Mixed methods	Interprofessional	Psychiatric	Multiple	Reach, Effectiveness, Adoption, Implementation, and Maintenance (RE-AIM)	Evaluation framework	Evaluation of process<br>Evaluation of implementation outcomes<br>Evaluation of intervention outcomes<br>Identify barriers and facilitators
**Rafferty et al.<br>2023,<br>USA**	Mixed methods	Multiperspective	Not reported	Inpatient	Consolidated Framework for Implementation Research (CFIR)<br>Expert Recommendations for Implementation Change (ERIC)<br>Implementation Research Logic Model<br>Proctor's taxonomy of implementation outcomes	Determinant framework<br>Implementation theory<br>Evaluation framework	Evaluation of implementation oucomes

**Rafferty et al. 2019, USA**	Case report	Multiperspective	Neurological (movement disorders, including Parkinson's disease)	Outpatient	Consolidated Framework for Implementation Research (CFIR)<br>Knowledge-to-Action (KTA)<br>Reach, Effectiveness, Adoption, Implementation, and Maintenance (RE-AIM)	Determinant framework<br>Process model<br>Evaluation framework	Evaluation of implementation outcomes<br>Evaluation of intervention outcomes<br>Identify barriers and facilitators<br>Identify IS strategies<br>Specify or describe process of implementation
**Rivard ym. 2015, Canada**	Mixed methods	One Profession	Paediatric	Multiple	Knowledge-to-Action (KTA)	Process model	Development of IS strategies
**Romney et al. 2022a, USA**	Mixed methods	One Profession	Other (orthopedic)	Inpatient	Consolidated Framework for Implementation Research (CFIR)	Determinant framework	Evaluation of implementation outcomes<br>Identify barriers and facilitators
**Romney et al. 2022b, USA**	Quantitative	Other	Several	Inpatient	Knowledge-to-Action (KTA)	Process model	Evaluation of implementation outcomes
**Romney et al. 2019, USA**	Case report	One Profession	Several	Inpatient	Theoretical Domains Framework (TDF)	Determinant framework	Development of IS strategies<br>Guide implementation planning<br>Identify barriers and facilitators
**Roos et al. 2021, Canada**	Qualitative	Other	Other (occupational health issues)	Community	Consolidated Framework for Implementation Research (CFIR)<br>Theoretical Domains Framework (TDF)	Determinant framework	Identify barriers and facilitators
**Russell et al. 2010, Canada**	Mixed methods	One Profession	Paediatric	Multiple	Knowledge-to-Action (KTA)	Process model	Describe current practice<br>Evaluation of implementation outcomes<br>Specify relationship between constructs or mechanism

**Sabus & Spake 2016, USA**	Qualitative	One Profession (including participants in leadership positions)	Several	Outpatient	Consolidated Framework for Implementation Research (CFIR)	Determinant framework	Identify barriers and facilitators
**Sakzewski et al. 2014, Australia**	Qualitative	One Profession (including participants in leadership positions)	Paediatric	Multiple	Theoretical Domains Framework (TDF)	Determinant framework	Identify barriers and facilitators
**Salbach et al. 2021, Canada**	Qualitative	Interprofessional (including participants in leadership positions)	Neurological (stroke)	Multiple	Theoretical Domains Framework (TDF)	Determinant framework	Specify relationship between constructs or mechanism
**Salbach et al. 2017, Canada**	Quantitative	Multiperspective	Neurological (stroke)	Inpatient	Knowledge-to-Action (KTA)	Process model	Evaluation of implementation outcomes<br>Evaluation of intervention outcomes
**Schell et al. 2020, Canada**	Qualitative	Interprofessional	Paediatric	Multiple	Knowledge-to-Action (KTA)	Process model	Specify or describe process of implementation
**Schliep et al. 2020, USA**	Quantitative	One Profession	Aphasia	Inpatient	Quality Implementation Framework (QIF)	Process model	Evaluation of implementation outcomes<br>Evaluation of intervention outcomes<br>Specify or describe process of implementation
**Schreiber et al. 2015, USA**	Case report	One Profession	Paediatric	Outpatient	Knowledge-to-Action (KTA)	Process model	Evaluation of implementation outcomes<br>Specify or describe process of implementation

**Schwarz et al. 2022, Australia**	Qualitative	Interprofessional	Other (dysphagia)	Inpatient	Consolidated Framework for Implementation Research (CFIR)	Determinant framework	Identify barriers and facilitators
**Shafer et al. 2023, USA**	Qualitative	Multiperspective	Aphasia	Multiple	Knowledge-to-Action (KTA)	Process model	Identify barriers and facilitators
**Sheppard et al. 2022, Canada**	Qualitative	Multiperspective	Other (covid-19)	Inpatient	Consolidated Framework for Implementation Research (CFIR)	Determinant framework	Identify barriers and facilitators
**Shrubsole 2021, Australia**	Mixed methods	Interprofessional	Neurological (movement disorders, including Parkinson's disease)	Outpatient	Knowledge-to-Action (KTA)	Process model	Evaluation of implementation outcomes<br>Identify barriers and facilitators<br>Specify or describe process of implementation
**Shrubsole et al. 2019, Australia**	Qualitative	One Profession	Aphasia	Inpatient	Theoretical Domains Framework (TDF)	Determinant framework	Describe current practice<br>Identify barriers and facilitators
**Sibley & Salbach 2015, Canada**	Case report	One Profession	Several	Not reported	Knowledge-to-Action (KTA)	Process model	Specify or describe process of implementation
**Sohlberg et al. 2015, USA**	Mixed methods	Other	Neurological (brain injury)	Multiple	Consolidated Framework for Implementation Research (CFIR)	Determinant framework	Specify or describe process of implementation<br>Development or adaptation of intervention
**Song et al. 2018, Australia**	Mixed methods	Clients	Several	Inpatient	Reach, Effectiveness, Adoption, Implementation, and Maintenance (RE-AIM)	Evaluation framework	Evaluation of implementation outcomes<br>Evaluation of intervention outcomes

							Evaluation of process<br>Identify barriers and facilitators
**Stewart et al. 2020, Australia**	Mixed methods	Interprofessional	Neurological (stroke)	Inpatient	Behaviour Change wheel (BCW)	Implementation theory	Development of IS strategies<br>Identify barriers and facilitators
**Stout et al. 2024, USA**	Qualitative	Multiperspective (including participants in leadership positions)	Cancer	Outpatient	Consolidated Framework for Implementation Research (CFIR)<br>Expert Recommendations for Implementation Change (ERIC)	Determinant framework	Identify barriers and facilitators<br>Identify IS strategies
**Stout et al. 2023, USA**	Qualitative	Interprofessional	Cancer	Outpatient	Consolidated Framework for Implementation Research (CFIR)<br>Expert Recommendations for Implementation Change (ERIC)	Determinant framework	Identify barriers and facilitators<br>Evaluation of implementation outcomes
**Tierney-Hendricks et al. 2022, USA**	Quantitative	One Profession	Aphasia	Multiple	Consolidated Framework for Implementation Research (CFIR)	Determinant framework	Describe current practice
**Trebilcock et al. 2022, Australia**	Qualitative	One Profession	Aphasia	Not reported	Behaviour Change wheel (BCW)<br>Integrated Knowledge Translation (IKT)	Implementation theory<br>Process model	Development of IS strategies
**Trebilcock et al. 2024, Australia**	Qualitative	One Profession	Aphasia	Multiple	Recommendations for the development, implementation, evaluation, and reporting of online KT resources	Evaluation framework	Evaluation of implementation outcomes

**Trebilcock et al. 2023, Australia**	Mixed methods	One Profession	Aphasia	Multiple	Theoretical Framework of Acceptability (TFA)	Evaluation framework	Evaluation of implementation outcomes<br>Guide implementation planning
**Van Der Veen et al. 2019, Netherlands**	Qualitative	Interprofessional (including participants in leadership positions)	Neurological (stroke)	Home-based	Consolidated Framework for Implementation Research (CFIR)	Determinant framework	Describe current practice<br>Identify barriers and facilitators
**Van Grootven et al. 2022, Belgium**	Mixed methods	Multiperspective	Geriatric	Inpatient	Grol's and Wensing's model	Process model	Evaluation of implementation outcomes<br>Identify barriers and facilitators
**Van Stan et al. 2023, USA**	Qualitative	Interprofessional	Several	Multiple	Consolidated Framework for Implementation Research (CFIR)<br>Expert Recommendations for Implementation Change (ERIC)	Determinant framework	Identify barriers and facilitators<br>Identify IS strategies
**Van Twillert et al. 2015, Netherlands**	Qualitative	Multiperspective (including participants in leadership positions)	Other (amputation)	Multiple	Knowledge-to-Action (KTA)	Process model	Specify or describe process of implementation
**Vinzenco et al. 2024, USA**	Quantitative	Other	Geriatric	Outpatient	Reach, Effectiveness, Adoption, Implementation, and Maintenance (RE-AIM)	Evaluation framework	Evaluation of implementation outcomes<br>Specify or describe process of implementation
**Wakefield et al. 2019, USA**	Mixed methods	Multiperspective (including participants in leadership positions)	Cardiovascular	Home-based	Consolidated Framework for Implementation Research (CFIR)	Determinant framework	Identify barriers and facilitators<br>Specify relationship between constructs or mechanism

**Walsh et al. 2022, USA**	Qualitatative	Other	Neurological (spinal cord injury)	Inpatient	Collaborative Intervention Planning Framework (CIPF)	Process model	Development or adaptation of intervention<br>Guide Implementation planning
**Wang et al. 2023, China**	Qualitative	One Profession	Geriatric	Home-based	Consolidated Framework for Implementation Research (CFIR)	Determinant framework	Identify barriers and facilitators
**Wintner et al. 2021, Austria**	Qualitative	Multiperspective	Cancer	Inpatient	The replicating effective programmes framework (REP)	Process model	Specify or describe process of implementation
**Wong et al. 2023, USA**	Mixed methods	Multiperspective	Neurological (stroke)	Home-based	Behaviour Change Taxonomy<br>Intervention mapping (IM)	Implementation theory<br>Process model	Development or adaptation of intervention
**Yang et al. 2021, Canada**	Mixed methods	Multiperspective	Neurological (stroke)	Community	Reach, Effectiveness, Adoption, Implementation, and Maintenance (RE-AIM)	Evaluation framework	Evaluation of process<br>Evaluation of implementation outcomes<br>Evaluation of intervention outcomes
**Yorke et al. 2021, USA**	Mixed methods	Interprofessional	Neurological (movement disorders, including Parkinson's disease)	Outpatient	Knowledge-to-Action (KTA)	Process model	Development of IS strategy<br>Evaluation of process<br>Evaluation of implementation outcomes<br>Evaluation of intervention outcomes
**Young et al. 2018, Australia**	Quantitative	One Profession	Aphasia	Multiple	Theoretical Domains Framework (TDF)	Determinant framework	Identify barriers and facilitators<br>Specify relationship between constructs or mechanism

Appendix S3. References of the included studies (n = 121)

1. Adsul P, Schmitz K, Basen-Engquist KM, Rogers LQ. Studying the implementation of exercise oncology interventions: a path forward. Transl J Am Coll Sports Med 2022; 7:1–8. doi:10.1249/tjx.0000000000000208

2. Ahmed S, Zidarov D, Eilayyan O, Visca R. Prospective application of implementation science theories and frameworks to inform use of PROMs in routine clinical care within an integrated pain network. Qual Life Res 2021; 30: 3035–3047. doi:10.1007/s11136-020-02600-8

3. Alatawi SF. From theory to practice: a conceptual framework to facilitate implementation of evidence in stroke rehabilitation for local context in Saudi Arabia. J Multidiscip Healthc 2019; 12: 515–525. doi:10.2147/JMDH.S212372

4. Alatawi SF. How can we use the Promoting Action on Research in Health Services (PARIHS) framework to move from what we know to what we should do for the rehabilitation of a painful hemiplegic shoulder (PHS)? J Multidiscip Healthc 2022; 15: 2831–2843. doi:10.2147/JMDH.S392376

5. Allen KM, Dittmann KR, Hutter JA, Chuang C, Donald ML, Enns AL, et al. Implementing a shared decision-making and cognitive strategy-based intervention: knowledge user perspectives and recommendations. J Eval Clin Pract 2020; 26: 575–581. doi:10.1111/jep.13329

6. Arnold H, Wallace SJ, Ryan B, Finch E, Shrubsole K. Current practice and barriers and facilitators to outcome measurement in aphasia rehabilitation: a cross-sectional study using the theoretical domains framework. Aphasiology 2020; 34: 47–69. doi:10.1080/02687038.2019.1678090

7. Auld ML, Johnston LM. Getting inTOUCH: outcomes of a knowledge translation intervention for tactile assessment knowledge, barriers, and practice in paediatric therapists working with children with cerebral palsy. Disabil Rehabil 2019; 41: 2350–2358. doi:10.1080/09638288.2018.1466202

8. Bird ML, Mortenson BW, Chu F, Acerra N, Bagnall E, Wright A, et al. Building a bridge to the community: an integrated knowledge translation approach to improving participation in community-based exercise for people after stroke. Phys Ther 2019; 99: 286–296. doi:10.1093/ptj/pzy146

9. Bond GR, Johnson-Kwochka AV, Pogue JA, Langfitt Reese S, Becker DR, Drake RE. A tale of four states: factors influencing the statewide adoption of IPS. Adm Policy Ment Health 2021; 48: 528–538. doi:10.1007/s10488-020-01087-2

10. Bozec E, Gorska J, Grampurohit N. Upper extremity task-specific training: manual development and implementation research within inpatient rehabilitation. Occup Ther Health Care 2021; 35: 336–354. doi:10.1080/07380577.2021.1938338

11. Brouns B, Meesters JJL, Wentink MM, de Kloet AJ, Arwert HJ, Vliet Vlieland TPM, et al. Why the uptake of eRehabilitation programs in stroke care is so difficult: a focus group study in the Netherlands. Implement Sci 2018; 13: 133. doi:10.1186/s13012-018-0827-5

12. Cahill LS, Carey LM, Mak-Yuen Y, McCluskey A, Neilson C, O'Connor DA, et al. Factors influencing allied health professionals' implementation of upper limb sensory rehabilitation for stroke survivors: a qualitative study to inform knowledge translation. BMJ Open 2021; 11: e042879. doi:10.1136/bmjopen-2020-042879

13. Camden C, Rivard L, Pollock N, Missiuna C. Knowledge to practice in developmental coordination disorder: impact of an evidence-based online module on physical therapists' self-reported knowledge, skills, and practice. Phys Occup Ther Pediatr 2015; 35: 195–210. doi:10.3109/01942638.2015.1012318

14. Celian C, Swanson V, Shah M, Newman C, Fowler-King B, Gallik S, et al. A day in the life: a qualitative study of clinical decision-making and uptake of neurorehabilitation technology. J Neuroeng Rehabil 2021; 18:121. doi:10.1186/s12984-021-00911-6

15. Clare L, Kudlicka A, Collins R, Evans S, Pool J, Henderson C, et al. Implementing a home-based personalised cognitive rehabilitation intervention for people with mild-to-moderate dementia: GREAT into Practice. BMC Geriatr 2023; 23: 93. doi:10.1186/s12877-022-03705-0

17. Connell LA, McMahon NE, Watkins CL, Eng JJ. Therapists' use of the graded repetitive arm supplementary program (GRASP) intervention: a practice implementation survey study. Phys Ther 2014; 94: 632–643. doi:10.2522/ptj.20130240

18. Connell LA, McMahon NE, Tyson SF, Watkins CL, Eng JJ. Case series of a knowledge translation intervention to increase upper limb exercise in stroke rehabilitation. Phys Ther 2016; 96: 1930–1937. doi:10.2522/ptj.20150694

19. Connell LA, McMahon NE, Tyson SF, Watkins CL, Eng JJ. Mechanisms of action of an implementation intervention in stroke rehabilitation: a qualitative interview study. BMC Health Serv Res 2016; 16: 534. doi:10.1186/s12913-016-1793-8

20. Connell LA, Chesworth B, Ackerley S, Smith MC, Stinear CM. Implementing the PREP2 algorithm to predict upper limb recovery potential after stroke in clinical practice: a qualitative study. Phys Ther 2021; 101: pzab040. doi:10.1093/ptj/pzab040

21. Corbin S, Damiolini E, Termoz A, Huchon L, Rode G, Schott AM, et al. Rehabilitation professionals' views on individual peer support interventions for assisting stroke survivors with reintegration into the community: a qualitative study. Disabil Rehabil 2023; 45: 4413–4423. doi:10.1080/09638288.2022.2152115

22. Cox NS, Scrivener K, Holland AE, Jolliffe L, Wighton A, Nelson S, et al. A brief intervention to support implementation of telerehabilitation by community rehabilitation services during COVID-19: a feasibility study. Arch Phys Med Rehabil 2021; 102: 789–795. doi:10.1016/j.apmr.2020.12.007

23. Cunningham BJ, Rosenbaum P, Hidecker MJC. Promoting consistent use of the Communication Function Classification System (CFCS). Disabil Rehabil 2016; 38: 195–204. doi:10.3109/09638288.2015.1027009

24. Cunningham BJ, Hidecker MJC, Thomas-Stonell N, Rosenbaum P. Moving research tools into practice: the successes and challenges in promoting uptake of classification tools. Disabil Rehabil 2018; 40:1099–1107. doi:10.1080/09638288.2017.1280544

25. Daoust G, Rushton PW, Racine M, Leduc K, Assila N, Demers L. Adapting the Wheelchair Skills Program for pediatric rehabilitation: recommendations from key stakeholders. BMC Pediatr 2021; 21:103. doi:10.1186/s12887-021-02564-9

26. Demers M, Thomas A, Wittich W, McKinley P. Implementing a novel dance intervention in rehabilitation: perceived barriers and facilitators. Disabil Rehabil 2015; 37: 1066–1072. doi:10.3109/09638288.2014.955135

27. Déry J, Ruiz A, Routhier F, Gagnon MP, Côté A, Ait-Kadi D, et al. Barriers and facilitators for implementation of a patient prioritization tool in two specialized rehabilitation programs. JBI Evid Implement 2021; 19: 149–161. doi:10.1097/XEB.0000000000000281

28. Duran AT, Keener-DeNoia A, Stavrolakes K, Fraser A, Blanco LV, Fleisch E, et al. Applying user-centered design and implementation science to the early-stage development of a telehealth-enhanced hybrid cardiac rehabilitation program: quality improvement study. JMIR Form Res 2023; 7: e47264. doi:10.2196/47264

29. D'Souza S, Ciccone N, Hersh D, Janssen H, Armstrong E, Godecke E. Staff and volunteers' perceptions of a Communication Enhanced Environment model in an acute/slow stream rehabilitation and a rehabilitation hospital ward: a qualitative description study within a before-and-after pilot study. Disabil Rehabil 2022; 44: 7009–7022. doi:10.1080/09638288.2021.1977397

30. Eakin MN, Ugbah L, Arnautovic T, Parker AM, Needham DM. Implementing and sustaining an early rehabilitation program in a medical intensive care unit: a qualitative analysis. J Crit Care 2015; 30: 698–704. doi:10.1016/j.jcrc.2015.03.019

31. Fehlings MG, Cheng CL, Chan E, Thorogood NP, Noonan VK, Ahn H, et al. Using evidence to inform practice and policy to enhance the quality of care for persons with traumatic spinal cord injury. J Neurotrauma 2017; 34: 2934–2940. doi:10.1089/neu.2016.4938

32. Ferro AM, Quinn L. A structured goal-setting process to promote functional and measurable outcomes in school-based physical therapy: a knowledge translation study. Pediatr Phys Ther 2020; 32: 211–217. doi:10.1097/PEP.0000000000000707

33. Frost J, Wingham J, Britten N, Greaves C, Abraham C, Warren FC, et al. The value of social practice theory for implementation science: learning from a theory-based mixed methods process evaluation of a randomised controlled trial. BMC Med Res Methodol 2020; 20: 181. doi:10.1186/s12874-020-01060-5

34. Gaid D, Ahmed S, Thomas A, Bussières A. Barriers and facilitators to knowledge brokering activities: perspectives from knowledge brokers working in Canadian rehabilitation settings. J Contin Educ Health Prof 2023; 43: 87–95. doi:10.1097/CEH.0000000000000475

35. Giurleo C, McIntyre A, Kras-Dupuis A, Wolfe DL. Addressing the elephant in the room: integrating sexual health practice in spinal cord injury rehabilitation. Disabil Rehabil 2022; 44: 3245–3252. doi:10.1080/09638288.2020.1856949

36. Gorzelitz JS, Bouji N, Stout NL. Program barriers and facilitators in virtual cancer exercise implementation: a qualitative analysis. Transl J Am Coll Sports Med 2022; 7: e000199. doi:10.1249/tjx.0000000000000199

37. Grimmer K, Louw Q, Dizon JM, Brown SM, Ernstzen D, Wiysonge CS. A South African experience in applying the Adopt-Contextualise-Adapt framework to stroke rehabilitation clinical practice guidelines. Health Res Policy Syst 2019; 17: 56. doi:10.1186/s12961-019-0454-x

38. Hadely KA, Power E, O'Halloran R. Speech pathologists' experiences with stroke clinical practice guidelines and the barriers and facilitators influencing their use: a national descriptive study. BMC Health Serv Res 2014; 14: 110. doi:10.1186/1472-6963-14-110

39. Heinemann AW, Nitsch KP, Gracz K, Ehrlich-Jones L, Engel E, Wilson M, et al. Implementing patient-reported outcome measures in inpatient rehabilitation: challenges and solutions. Arch Phys Med Rehabil 2022; 103: S67–S77. doi:10.1016/j.apmr.2021.05.010

40. Hirschhorn AD, Kolt GS, Brooks AJ. Barriers and enablers to the provision and receipt of preoperative pelvic floor muscle training for men having radical prostatectomy: a qualitative study. BMC Health Serv Res 2013; 13: 305. doi:10.1186/1472-6963-13-305

41. Holmlund L, Hellman T, Engblom M, Kwak L, Sandman L, Törnkvist L, et al. Coordination of return-to-work for employees on sick leave due to common mental disorders: facilitators and barriers. Disabil Rehabil 2022; 44: 3113–3121. doi:10.1080/09638288.2020.1855263

42. Hreha K, Barrett AM, Gillen RW, Gonzalez-Snyder C, Masmela J, Chen P. The implementation process of two evidence-based protocols: a spatial neglect network initiative. Front Health Serv 2022; 2: 839517. doi:10.3389/frhs.2022.839517

43. Imms C, Kerr C, Bowe SJ, Karlsson P, Novak I, Shields N, et al; Best Service Best Time Author Group. Efficacy of a knowledge translation approach in changing allied health practitioner use of evidence-based practices with children with cerebral palsy: a before and after longitudinal study. Disabil Rehabil 2021; 43: 3592–3605. doi:10.1080/09638288.2020.1727576

44. Inness EL, Jagroop D, Andreoli A, Bayley M, Biasin L, Danells C, et al. Factors that influence the clinical implementation of aerobic exercise in stroke rehabilitation: a theory-informed qualitative study. Phys Ther 2022; 102: pzac014. doi:10.1093/ptj/pzac014

45. Jeong Y, Law M, DeMatteo C, Stratford P, Kim H. Knowledge translation from research to clinical practice: measuring participation of children with disabilities. Occup Ther Health Care 2016; 30: 323–343. doi:10.1080/07380577.2016.1192311

46. Jervis Rademeyer H, Gauthier C, Zariffa J, Walden K, Jeji T, McCullum S, et al. Using activity-based therapy for individuals with spinal cord injury or disease: interviews with physical and occupational therapists in rehabilitation hospitals. J Spinal Cord Med 2023;46:298–308. doi:10.1080/10790268.2022.2039855

47. Johanson S, Markström U, Larsson ME, Bejerholm U. Implementation of a novel return-to-work approach for persons with affective disorders in a traditional vocational rehabilitation context: a case study. Int J Ment Health Syst 2020;14:22. doi:10.1186/s13033-020-00355-w

48. Johnston V, Brakenridge C, Valiant D, Ling CLK, Andrews N, Gane EM, et al. Using framework analysis to understand multiple stakeholders' views of vocational rehabilitation following acquired brain injury. Brain Impair 2023; 24: 347–370. doi:10.1017/BrImp.2022.27

49. Jolliffe L, Andrew NE, Srikanth V, Beare R, Noeske KE, Snowdon DA. Development of an implementation strategy for routine collection of generic patient reported outcome measures: a qualitative study in multidisciplinary community rehabilitation. Disabil Rehabil 2024; 46: 3895–3904. doi:10.1080/09638288.2023.2258334

50. Jones C, Smith-MacDonald L, Brémault-Phillips S. Perceptions of Canadian Armed Forces Health Services health care professionals on cognitive assessment processes. J Mil Veteran Fam Health 2021; 7: 6–19. doi:10.3138/jmvfh-2020-0066

51. Kang E, Foster ER. Use of implementation mapping with community-based participatory research: development of implementation strategies of a new goal setting and goal management intervention system. Front Public Health 2022; 10: 834473. doi:10.3389/fpubh.2022.834473

52. Kang E, Chen J, Foster ER. Implementation strategies for occupational therapists to advance goal setting and goal management. Front Health Serv 2023; 3: 1042029. doi:10.3389/frhs.2023.1042029

53. Karlsson I, Kwak L, Axén I, Bergström G, Bültmann U, Holmgren K, et al. Experiences of participating in a problem-solving intervention with workplace involvement in Swedish primary health care: a qualitative study from rehabilitation coordinator's, employee's, and manager's perspectives. BMC Public Health 2023; 23: 940. doi:10.1186/s12889-023-15899-y

54. Kelly G, Moys R, Burrough M, Hyde S, Randall S, Wales L. Rehabilitation in practice: improving delivery of upper limb rehabilitation for children and young people with acquired brain injuries through the development and implementation of a clinical pathway. Disabil Rehabil 2022; 44: 158–165. doi:10.1080/09638288.2020.1761891

55. Kettlewell J, Radford K, Kendrick D, Patel P, Bridger K, Kellezi B, et al; ROWTATE Team. Qualitative study exploring factors affecting the implementation of a vocational rehabilitation intervention in the UK major trauma pathway. BMJ Open 2022; 12: e060294. doi:10.1136/bmjopen-2021-060294

56. Kengne Talla P, Robillard C, Ahmed S, Guindon A, Houtekier C, Thomas A. Clinical research coordinators' role in knowledge translation activities in rehabilitation: a mixed methods study. BMC Health Serv Res 2023; 23: 124. doi:10.1186/s12913-023-09027-0

57. Kim E, Lee M, Kim EH, Kim HJ, Koo M, Cheong IY, et al. Using knowledge translation to establish a model of hospital-based early supported community reintegration for stroke patients in South Korea. BMC Health Serv Res 2021; 21: 1359. doi:10.1186/s12913-021-07400-5

58. Kingsnorth S, Orava T, Parker K, Milo-Manson G. From knowledge translation theory to practice: developing an evidence to care hub in a pediatric rehabilitation setting. Disabil Rehabil 2020; 42: 869–879. doi:10.1080/09638288.2018.1514075

59. Kristensen HK, Hounsgaard L. Implementation of coherent, evidence-based pathways in Danish rehabilitation practice. Disabil Rehabil 2013; 35: 2021–2028. doi:10.3109/09638288.2013.768301

60. Lamper C, Huijnen IPJ, Kroese MEAL, Köke AJ, Brouwer G, Ruwaard D, et al. Exploring the feasibility of a network of organizations for pain rehabilitation: what are the lessons learned? PLoS One 2022; 17: e0273030. doi:10.1371/journal.pone.0273030

61. Levy T, Killington M, Laver K, Lannin NA, Crotty M. Developing and implementing an exercise-based group for stroke survivors and their carers: the Carers Count group. Disabil Rehabil 2022; 44: 3982–3991. doi:10.1080/09638288.2021.1897693

62. Lundquist CB, Pallesen H, Tjørnhøj-Thomsen T, Brunner IC. Exploring physiotherapists' and occupational therapists' perceptions of the upper limb prediction algorithm PREP2 after stroke in a rehabilitation setting: a qualitative study. BMJ Open 2021; 11: e038880. doi:10.1136/bmjopen-2020-038880

63. Lynch EA, Connell LA, Carvalho LB, Bird ML. Do clinical guidelines guide clinical practice in stroke rehabilitation? An international survey of health professionals. Disabil Rehabil 2022; 44: 4118–4125. doi:10.1080/09638288.2021.1891304

64. Mayo AL, Fung V, Hitzig SL, Gould S, Posa S, Summers deLuca L, et al. Exploring the psychosocial needs of persons with lower extremity amputation and feasibility of internet cognitive behavioural therapy: a qualitative study. Disabil Rehabil 2023; 45: 4025–4034. doi:10.1080/09638288.2022.2144492

65. McArthur C, Ziebart C, Papaioannou A, Cheung AM, Laprade J, Lee L, et al. “We get them up, moving, and out the door. How do we get them to do what is recommended?” Using behaviour change theory to put exercise evidence into action for rehabilitation professionals. Arch Osteoporos 2018; 13:7. doi:10.1007/s11657-018-0419-7

66. McEwen SE, Donald M, Jutzi K, Allen KA, Avery L, Dawson DR, et al. Implementing a function-based cognitive strategy intervention within inter-professional stroke rehabilitation teams: changes in provider knowledge, self-efficacy and practice. PLoS One 2019; 14: e0212988. doi:10.1371/journal.pone.0212988

67. Mc Sharry J, Murphy PJ, Byrne M. Implementing international sexual counselling guidelines in hospital cardiac rehabilitation: development of the CHARMS intervention using the Behaviour Change Wheel. Implement Sci 2016; 11: 134. doi:10.1186/s13012-016-0493-4

68. Miao M, Morrow R, Salomon A, McCulloch B, Evain JC, Wright MR, et al. Digital health implementation strategies coproduced with adults with acquired brain injury, their close others, and clinicians: mixed methods study with collaborative autoethnography and network analysis. J Med Internet Res 2023; 25: e46396. doi:10.2196/46396

69. Moore JL, Virva R, Henderson C, Lenca L, Butzer JF, Lovell L, et al. Applying the Knowledge-to-Action framework to implement gait and balance assessments in inpatient stroke rehabilitation. Arch Phys Med Rehabil 2022; 103: S230–S245. doi:10.1016/j.apmr.2020.10.133

70. Moore JL, Carpenter J, Doyle AM, Doyle L, Hansen P, Hahn B, et al. Development, implementation, and use of a process to promote knowledge translation in rehabilitation. Arch Phys Med Rehabil 2018; 99: 82–90. doi:10.1016/j.apmr.2017.08.476

71. Moore JL, Bø E, Erichsen A, Rosseland I, Halvorsen J, Bratlie H, et al. Development and results of an implementation plan for high-Intensity gait training. J Neurol Phys Ther 2021; 45: 282–291. doi:10.1097/NPT.0000000000000364

72. Munce SEP, Graham ID, Salbach NM, Jaglal SB, Richards CL, Eng JJ, et al. Perspectives of health care professionals on the facilitators and barriers to the implementation of a stroke rehabilitation guidelines cluster randomized controlled trial. BMC Health Serv Res 2017; 17: 440. doi:10.1186/s12913-017-2389-7

73. Nitsch KP, Stipp K, Gracz K, Ehrlich-Jones L, Graham ID, Heinemann AW. Integrating spinal cord injury–quality of life instruments into rehabilitation: implementation science to guide adoption of patient-reported outcome measures. J Spinal Cord Med 2021; 44: 940–948. doi:10.1080/10790268.2020.1712893

74. Orava T, Provvidenza C, Townley A, Kingsnorth S. Screening and assessment of chronic pain among children with cerebral palsy: a process evaluation of a pain toolbox. Disabil Rehabil 2019; 41: 2695–2703. doi:10.1080/09638288.2018.1471524

75. Pastva AM, Coyle PC, Coleman SW, Radman MD, Taylor KM, Jones SB, et al; COMPASS Investigative Team. Movement Matters, and So Does Context: lessons learned from multisite implementation of the Movement Matters Activity Program for Stroke in the Comprehensive Postacute Stroke Services Study. Arch Phys Med Rehabil 2021; 102: 532–542. doi:10.1016/j.apmr.2020.09.386

76. Patterson K, Keegan R, Davey R, Freene N. Implementing a sedentary behavior change smartphone app in cardiac rehabilitation: a qualitative analysis guided by the Theoretical Domains Framework and Capability, Opportunity, and Motivation-Behavior Model. J Cardiovasc Nurs 2024; 39:e12–e20. doi:10.1097/JCN.0000000000000983

77. Pepin ME, Astronomo R, Brown A, Fritz NE. Standardized screening of cognitive and affective tendencies in persons with low back pain: a knowledge translation project. Physiother Theory Pract 2024; 40: 1961–1973. doi:10.1080/09593985.2023.2229902

78. Perez C, Kaizer F, Archambault P, Fung J. A novel approach to integrate VR exer-games for stroke rehabilitation: evaluating the implementation of a “games room”. In: Proc Int Conf Virtual Rehabil (ICVR); 2017; Montreal, QC, Canada. p. 1–7. doi:10.1109/ICVR.2017.8007538

79. Pittman JOE, Davidson EJ, Dozier ME, Blanco BH, Baer KA, Twamley EW, et al. Implementation and evaluation of a community-based treatment for late-life hoarding. Int Psychogeriatr 2021; 33: 977–986. doi:10.1017/S1041610220000241

80. Rafferty MR, MacDonald J, Byskosh A, Sloan L, Toledo S, Marciniak C, et al. Using Implementation Frameworks to Provide Proactive Physical Therapy for People With Parkinson Disease: case report. Phys Ther 2019; 99: 1644–1655. doi:10.1093/ptj/pzz129

81. Rafferty M, Stoff L, Smith JD, Hansen P, Briody M, Diaz C, et al. Promoting Evidence-Based Practice: the influence of novel structural change to accelerate translational rehabilitation. Arch Phys Med Rehabil 2023; 104: 1289–1299. doi:10.1016/j.apmr.2023.02.014

82. Rivard L, Camden C, Pollock N, Missiuna C. Knowledge to practice in developmental coordination disorder: utility of an evidence-based online module for physical therapists. Phys Occup Ther Pediatr 2015; 35: 178–194. doi:10.3109/01942638.2014.985414

83. Romney W, Salbach N, Parrott JS, Deutsch JE. A knowledge translation intervention designed using audit and feedback and the Theoretical Domains Framework for physical therapists working in inpatient rehabilitation: a case report. Physiother Theory Pract 2019; 35: 686–702. doi:10.1080/09593985.2018.1457113

84. Romney W, Salbach NM, Parrott JS, Ward IG, Deutsch JE. A knowledge broker facilitated intervention to improve the use of standardized assessment tools by physical therapists: a cluster randomized trial. Clin Rehabil 2022; 36: 214–229. doi:10.1177/02692155211046460

85. Romney W, Wormley M, Veneri D, Oberlander A, Grevelding P, Rice J, et al. Knowledge translation intervention increased the use of outcome measures by physical therapists in inpatient rehabilitation. Physiother Theory Pract 2022; 38: 2019–2028. doi:10.1080/09593985.2021.1898065

86. Roos M, Roy JS, Lamontagne ME. A qualitative study exploring the implementation determinants of rehabilitation and global wellness programs for orchestral musicians. Clin Rehabil 2021; 35: 1488–1499. doi:10.1177/02692155211010254

87. Russell DJ, Rivard LM, Walter SD, Rosenbaum PL, Roxborough L, Cameron D, et al. Using knowledge brokers to facilitate the uptake of pediatric measurement tools into clinical practice: a before-after intervention study. Implement Sci 2010; 5: 92. doi:10.1186/1748-5908-5-92

88. Sabus C, Spake E. Innovative physical therapy practice: a qualitative verification of factors that support diffusion of innovation in outpatient physical therapy practice. J Healthc Leadersh 2016; 8: 107–120. doi:10.2147/JHL.S115772

89. Sakzewski L, Ziviani J, Boyd RN. Delivering evidence-based upper limb rehabilitation for children with cerebral palsy: barriers and enablers identified by three pediatric teams. Phys Occup Ther Pediatr 2014; 34: 368–383. doi:10.3109/01942638.2013.861890

90. Salbach NM, Wood-Dauphinee S, Desrosiers J, Eng JJ, Graham ID, Jaglal SB, et al; SCORE-IT Team. Facilitated interprofessional implementation of a physical rehabilitation guideline for stroke in inpatient settings: process evaluation of a cluster randomized trial. Implement Sci 2017; 12: 100. doi:10.1186/s13012-017-0631-7

91. Salbach NM, McDonald A, MacKay-Lyons M, Bulmer B, Howe JA, Bayley MT, et al. Experiences of physical therapists and professional leaders with implementing a toolkit to advance walking assessment poststroke: a realist evaluation. Phys Ther 2021; 101: pzab232. doi:10.1093/ptj/pzab232

92. Schell S, Roth K, Duchow H. Developmental Coordination Disorder in Alberta: a journey into knowledge translation. Phys Occup Ther Pediatr 2020; 40: 294–310. doi:10.1080/01942638.2019.1664704

93. Schliep ME, Kasparian L, Kaminski O, Tierney-Hendricks C, Ayuk E, Brady Wagner L, et al. Implementing a standardized language evaluation in the acute phases of aphasia: linking evidence-based practice and practice-based evidence. Front Neurol 2020; 11: 412. doi:10.3389/fneur.2020.00412

94. Schreiber J, Marchetti GF, Racicot B, Kaminski E. The use of a knowledge translation program to increase use of standardized outcome measures in an outpatient pediatric physical therapy clinic: administrative case report. Phys Ther 2015; 95: 613–629. doi:10.2522/ptj.20130434

95. Schwarz M, Ward EC, Cornwell P, Coccetti A. Dysphagia screening using an allied health assistant delegation model: service considerations for implementation. Disabil Rehabil 2022; 44: 1275–1283. doi:10.1080/09638288.2020.1800109

96. Shafer JS, Haley KL, Jacks A. Barriers to informational support for care partners of people with aphasia after stroke. Am J Speech Lang Pathol 2023; 32: 2211–2231. doi:10.1044/2023_AJSLP-22-00391

97. Sheppard CL, Szigeti Z, Simpson R, Minezes J, Hitzig SL, Mayo A, et al. Implementation considerations for delivering inpatient COVID rehabilitation: a qualitative study. J Eval Clin Pract 2022; 28: 971–985. doi:10.1111/jep.13757

98. Shrubsole K. Implementation of an integrated multidisciplinary Movement Disorders Clinic: applying a knowledge translation framework to improve multidisciplinary care. Disabil Rehabil 2021; 43: 2071–2083. doi:10.1080/09638288.2019.1691666

99. Shrubsole K, Worrall L, Power E, O'Connor DA. Barriers and facilitators to meeting aphasia guideline recommendations: what factors influence speech pathologists' practice? Disabil Rehabil 2019; 41: 1596–1607. doi:10.1080/09638288.2018.1432706

100. Sibley KM, Salbach NM. Applying knowledge translation theory to physical therapy research and practice in balance and gait assessment: case report. Phys Ther 2015; 95: 579–587. doi:10.2522/ptj.20130486

101. Sohlberg MM, Kucheria P, Fickas S, Wade SL. Developing brain Injury interventions on both ends of the treatment continuum depends upon early research partnerships and feasibility studies. J Speech Lang Hear Res 2015; 58: S1864–S1870. doi:10.1044/2015_JSLHR-L-15-0150

102. Song K, Amatya B, Khan F. Advance care planning in rehabilitation: an implementation study. J Rehabil Med 2018; 50: 652–660. doi:10.2340/16501977-2356

103. Stewart C, Power E, McCluskey A, Kuys S. Development of a participatory, tailored behaviour change intervention to increase active practice during inpatient stroke rehabilitation. Disabil Rehabil 2020; 42: 3516–3524. doi:10.1080/09638288.2019.1597178

104. Stout NL, Utzman R, Jenkins HH, Burkart M, Swisher AK. Implementing and sustaining a breast cancer prospective surveillance rehabilitation program: an institutional perspective. J Cancer Surviv 2023; 17: 509–517. doi:10.1007/s11764-022-01304-x

105. Stout NL, Harrington SE, Perry A, Alappattu MJ, Pfab V, Stewart B, et al. Implementation of a cancer rehabilitation navigation program: a qualitative analysis of implementation determinants and strategies. J Cancer Surviv 2024; 18: 1325–1338. doi:10.1007/s11764-023-01374-5

106. Tierney-Hendricks C, Schliep ME, Vallila-Rohter S. Using an implementation framework to survey outcome measurement and treatment practices in aphasia. Am J Speech Lang Pathol 2022; 31: 1133–1162. doi:10.1044/2021_AJSLP-21-00101

107. Trebilcock M, Shrubsole K, Worrall L, Ryan B. Development of an online implementation intervention for aphasia clinicians to increase the intensity and comprehensiveness of their service. Disabil Rehabil 2022; 44: 4629–4638. doi:10.1080/09638288.2021.1910867

108. Trebilcock M, Shrubsole K, Worrall L, Ryan B. A survey of speech pathologists' opinions about the prospective acceptability of an online implementation platform for aphasia services. Int J Lang Commun Disord 2023; 58: 390–405. doi:10.1111/1460-6984.12796

109. Trebilcock M, Power E, Rose ML, Shrubsole K. How do speech pathologists use the Australian Aphasia Rehabilitation Pathway to inform practice? A qualitative study. Aphasiology 2024; 38: 957–978. doi:10.1080/02687038.2023.2246214

110. Van der Veen DJ, Döpp CME, Siemonsma PC, Nijhuis-van der Sanden MWG, de Swart BJM, Steultjens EM. Factors influencing the implementation of home-based stroke rehabilitation: professionals' perspective. PLoS One 2019; 14: e0220226. doi:10.1371/journal.pone.0220226

111. Van Grootven B, Jeuris A, Jonckers M, Devriendt E, Dierckx de Casterlé B, Dubois C, et al. How to implement geriatric co-management in your hospital? Insights from the G-COACH feasibility study. BMC Geriatr 2022; 22: 386. doi:10.1186/s12877-022-03051-1

112. Van Stan JH, Holmes J, Wengerd L, Juckett LA, Whyte J, Pinto SM, et al. Rehabilitation Treatment Specification System: identifying barriers, facilitators, and strategies for implementation in research, education, and clinical care. Arch Phys Med Rehabil 2023; 104: 562–568. doi:10.1016/j.apmr.2022.09.021

113. Van Twillert S, Postema K, Geertzen JHB, Lettinga AT. Incorporating self-management in prosthetic rehabilitation: case report of an integrated knowledge-to-action process. Phys Ther 2015; 95: 640–647. doi:10.2522/ptj.20130489

114. Vincenzo JL, Caulley J, Scott AJ, Wilson BS, Wingood M, Curran GM. Integrating STEADI for falls prevention in outpatient rehabilitation clinics: an outcomes evaluation using the RE-AIM framework. Gerontologist 2024; 64: gnad117. doi:10.1093/geront/gnad117

115. Wakefield BJ, Drwal K, Paez M, Grover S, Franciscus C, Reisinger HS, et al. Creating and disseminating a home-based cardiac rehabilitation program: experience from the Veterans Health Administration. BMC Cardiovasc Disord 2019; 19: 242. doi:10.1186/s12872-019-1224-y

116. Walsh RJ, McKay VR, Hansen PE, Barco PP, Jones K, Lee Y, et al. Using implementation science to guide the process of adapting a patient engagement intervention for inpatient spinal cord injury/disorder rehabilitation. Arch Phys Med Rehabil 2022; 103: 2180–2188. doi:10.1016/j.apmr.2022.04.010

117. Wang H, Zhang Y, Yue S. Exploring barriers to and facilitators of the implementation of home rehabilitation care for older adults with disabilities using the Consolidated Framework for Implementation Research (CFIR). BMC Geriatr 2023; 23: 292. doi:10.1186/s12877-023-03976-1

118. Wintner LM, Sztankay M, Riedl D, Rumpold G, Nickels A, Licht T, et al. How to implement routine electronic patient-reported outcome monitoring in oncology rehabilitation. Int J Clin Pract 2021; 75: e13694. doi:10.1111/ijcp.13694

119. Wong AWK, Fong MWM, Munsell EGS, Metts CL, Lee SI, Nicol GE, et al. Using intervention mapping and behavior change techniques to develop a digital intervention for self-management in stroke: development study. JMIR Hum Factors 2023; 10: e45099. doi:10.2196/45099

119. Yang CL, Bird ML, Eng JJ. Implementation and evaluation of the graded repetitive arm supplementary program (GRASP) for people with stroke in a real world community setting: case report. Phys Ther 2021; 101:pzab008. doi:10.1093/ptj/pzab008

120. Yorke AM, Trojanowski S, Fritz NE, Ludwa A, Schroeder M. Standardizing outcome assessment in Parkinson disease: a knowledge translation project. J Neurol Phys Ther 2021; 45: 21–27. doi:10.1097/NPT.0000000000000343

121. Young L, Shrubsole K, Worrall L, Power E. Factors that influence Australian speech-language pathologists' self-reported uptake of aphasia rehabilitation recommendations from clinical practice guidelines. Aphasiology 2018; 32: 646–665. doi:10.1080/02687038.2018.1443201
